# Antimicrobial and Immunomodulatory Effect of Gum Arabic on Human and Bovine Granulocytes Against *Staphylococcus aureus* and *Escherichia coli*

**DOI:** 10.3389/fimmu.2019.03119

**Published:** 2020-01-31

**Authors:** Shima Hassan Baien, Jana Seele, Timo Henneck, Christin Freibrodt, György Szura, Hani Moubasher, Roland Nau, Graham Brogden, Matthias Mörgelin, Mahavir Singh, Manfred Kietzmann, Maren von Köckritz-Blickwede, Nicole de Buhr

**Affiliations:** ^1^Department of Physiological Chemistry, Department of Infectious Diseases, University of Veterinary Medicine Hannover, Hanover, Germany; ^2^Research Center for Emerging Infections and Zoonoses (RIZ), University of Veterinary Medicine Hannover, Hanover, Germany; ^3^Department of Geriatrics, Evangelisches Krankenhaus Göttingen-Weende, Göttingen, Germany; ^4^Department of Neuropathology, University Medical Center Göttingen, Georg-August-University Göttingen, Göttingen, Germany; ^5^Clinic for Cattle, University of Veterinary Medicine Hannover, Hanover, Germany; ^6^Department of Botany and Microbiology, Faculty of Science, Cairo University, Cairo, Egypt; ^7^Colzyx AB, Medicon Village, Lund, Sweden; ^8^LIONEX Diagnostics and Therapeutics, GmbH, Brunswick, Germany; ^9^Department of Pharmacology, Toxicology and Pharmacy, University of Veterinary Medicine Hannover, Hanover, Germany

**Keywords:** gum arabic, neutrophils, immune modulation, indirect effect, direct effect, *Staphylococcus aureus*, *Escherichia coli*

## Abstract

Gum arabic (GA) is a traditional herbal medicine from *Acacia Senegal* (L.) Willdenow trees, which consist of a complex mixture of polysaccharides and glycoproteins. It is used in daily applications for several diseases and is considered to protect against bacterial infections. The detailed mechanisms behind these observations are still unclear. In this study, we investigated the direct antibacterial activity of GA water and ethanol extracts against *Staphylococcus* (*S*.) *aureus* or *Escherichia* (*E*.) *coli* and the immunomodulating properties of those extracts on granulocytes as a first line of defense against bacteria. Firstly, the direct antimicrobial effect of GA was tested on three different *S. aureus* strains and two *E. coli* strains. The growth of bacteria was analyzed in the presence of different GA concentrations over time. GA water as well as ethanol extracts showed a significant growth inhibition in a concentration-dependent manner in the case of *S. aureus* Newman, *S. aureus* Rd5, and *E. coli* 25922, but not in the case of *S*. *aureus* USA300 and *E. coli* K1. Transmission electron microscopic analysis confirmed an antibacterial effect of GA on the bacteria. Secondly, the immunomodulatory effect of GA on the antimicrobial activity of bovine or human blood-derived granulocytes was evaluated. Interestingly, water and ethanol extracts enhanced antimicrobial activity of granulocytes by the induction of intracellular ROS production. In line with these data, GA increased the phagocytosis rate of *E. coli*. No effect was seen on neutrophil extracellular trap (NET) formation that mediates killing of extracellular bacteria such as *S. aureus*. In conclusion, we show that GA exhibits a direct antibacterial effect against some *S. aureus* and *E. coli* strains. Furthermore, GA boosts the antimicrobial activities of granulocytes and increases intracellular ROS production, which may lead to more phagocytosis and intracellular killing. These data might explain the described putative antimicrobial activity of GA used in traditional medicine.

## Introduction

Gum arabic (GA) is a dried gummy exudate from stems and branches of *Acacia Senegal* (L.) Willdenow trees or closely related species. Chemically, GA is a water-soluble polysaccharide with sugars including rhamnose, arabinose, and galactose and contains highly branched complex arabinogalactan proteins (AGP). Furthermore, it contains glucuronic acid and minerals such as calcium, magnesium, and potassium (1–3).

GA is widely used as an emulsifier in the food industry and listed as a food additive (E414) by the European Food Safety Authority (EFSA) (4). Furthermore, GA has been shown to establish prebiotic efficacy (5). Another study shows that GA protects probiotic cultures of porcine gastric juice (6). In addition, GA has also been used as a traditional herbal medicine for hundreds of years in Africa. In this regard, GA has been reported to be used in traditional medicine for the treatment of inflammation of the intestinal mucosa, and to cover inflamed surfaces (7). In the Middle East and North Africa, GA is used as an oral hygiene substance that has anti-bacterial effects against periodontal pathogens (8). Furthermore, it has been tested for treatment of patients with chronic renal failure (9). A study conducted in Sudan demonstrated that the addition of GA to rats diet had a positive effect in lowering serum cholesterol and triacylglycerol (TAG) levels (10). In addition, a reduction of oxidative stress was shown after GA supplementation of drinking water in the liver tissue of rats with type I diabetes (11) and oral GA supplementation in humans suffering from sickle cell anemia (12). Altogether, it has been shown that GA influences the outcome of several different metabolism-related diseases or other non-infectious diseases [reviewed (13, 14)].

Some studies also report the influence of GA on infectious diseases. Ballal et al. demonstrated that GA has anti-malarial properties in plasmodium-infected mice, but with an unknown mechanism (15). The direct effect of GA against pathogenic bacteria such as *Staphylococcus* (*S*.) *aureus* and *Bacillus subtilis* was analyzed in the presence of milk products and *Lactobacillus*. A tendency to inhibit the growth was detected on agar plates and the strongest effect was shown for *S. aureus* growth inhibition in the presence of GA co-treated with *Lactobacillus plantarum* (16). In addition to those direct effects on bacteria, GA is considered as an effective additive in antimicrobial hydrogels for wound dressings [reviewed by (17)].

Moreover, an indirect effect of GA on bacteria by modulating immune cells was reported. The effect of GA on mouse dendritic cells was investigated by Xuan et al. (18), showing that the differentiation and maturation of bone marrow-derived dendritic cells as well as cytokine secretion were enhanced. On the other hand, the phagocytic activity of dendritic cells was decreased (18). An anti-inflammatory effect of GA was demonstrated via the application of GA in drinking water given to rats with diabetes mellitus by a decrease of TNFα and IL-1β and increase of IL-10 (19).

Interestingly, Bovo et al. (20) investigated the effect of GA on the complement system, as arabinogalactans are reported to be complement system modulators. The authors showed a pro-inflammatory activity of GA by activation of the classical and alternative pathways of the complement system (20). Thus, proinflammatory protective effects might be hypothesized for GA on innate immune cell function.

However, nothing is known about the effect of GA on granulocytes such as neutrophils, which are part of the first line of defense against bacterial infections. During the acute phase of a local inflammatory response, peripheral blood granulocytes, especially neutrophils, are the first responder cells to arrive in significant numbers and to fight against bacterial invaders.

Here, we investigated the antibacterial and immunomodulatory effect of a natural crude GA extract (NCE) and an ethanol precipitate (EP) from *Acacia Senegal* (L.) Willdenow trees from Sudan. To evaluate the effect for different species, we analyzed the effect of GA on the interaction between bovine and human blood-derived granulocytes and in response to different bovine and human isolates of *S. aureus* and *Escherichia* (*E*.) *coli*. Both pathogens can cause a diverse spectrum of diseases in human ranging from mild to severe invasive infections depending on the organ that is colonized. Furthermore, *S. aureus* and *E. coli* account for the majority of clinical mastitis cases in cattle and lead to high economic burden. Based on the emergence of antibiotic-resistant bacteria, deeper insight into alternative antibacterial and immunomodulatory strategies is needed.

## Materials and Methods

### GA Preparation

The natural GA was collected during summer 2015 from the Kordofan area in Western Sudan from *Acacia Senegal* (L.) Willdenow trees, family Mimosaceae. The *Acacia Senegal* was identified and taxonomically authenticated in the herbarium of Sudanese Medicinal and Aromatic Plants & Traditional Medicine Research Institute (MAPTRI) (Supplementary Figure 1). Commercial GA (CGA) from acacia trees was bought from Sigma-Aldrich as spray-dried powder.

Natural GA was cleaned from impurities by using a sharp object and then washed with sterile LPS-free water three times for 10 min. Finally, it was dissolved in LPS-free water with a concentration of 20% (w/v) and stirred with a magnetic stirrer at room temperature until it was completely dissolved. This solution was termed as natural crude extract (NCE) of GA. Further extraction was performed by addition of a 2-fold volume of 96% ethanol to 20% NCE of GA and incubated at −20°C for 24 h. Then, the solution was centrifuged at 15,000 × g for 20 min at 4°C to collect the EP. To maintain NCE in good quality by preventing degradation, the extract was dried in a freeze dryer machine. Freeze-dried samples were stored at 4°C as powder stocks.

NCE of GA and CGA were dissolved freshly in respective media prior to its usage for the different assays.

### Lipopolysaccharide (LPS) Detection

The degree of contamination with endotoxin of each extract was detected with Limulus amebocyte lysate (LAL) chromogenic kit (Thermo Fisher Scientific). The protocol was strictly followed as explained in the manufacturer's instructions. A standard curve was plotted by the average blank-corrected absorbance for each standard vs. its concentration in EU/ml. Endotoxin concentration of GA was determined based on the standard curve.

### Granulocyte Isolation

Venous blood from healthy cows was collected in K_3_EDTA tubes (Sarstedt) and the granulocytes were isolated using established protocols in our laboratory (21). Briefly, diluted blood was slowly added on the top of Biocoll (Merck Millipore; 1.077 g/ml) and then centrifuged at 1,100 × g for 30 min at 10°C. To harvest granulocytes from the pellet, red blood cells (RBCs) were lysed by using 0.2% NaCl for 30 s and lysis was stopped by adding 1.6% NaCl. The cells were centrifuged at 100 × g for 8 min at 4°C and resuspended in RPMI 1640 (Gibco).

Human granulocytes were isolated from fresh whole heparin blood of healthy donors using the Polymorph Prep system (Progen; 1.113 g/ml) as previously described (22).

### Cytotoxicity of GA

Bovine granulocytes (5 × 10^5^ cells /200 μl) were incubated with different concentrations of NCE starting from 0.0195 to 40 mg/ml at 37°C and atmospheric air supplemented with 5% CO_2_ for 30 min, 60 min and 120 min. As a positive control for dead cells, granulocytes treated with 100 μl buffer containing 0.2% Triton X-100 + 2% BSA in 1 × PBS for 15 min were used. Afterwards cells were stained with propidium iodide (Sigma-Aldrich; 1 mg/ml; 1:10) and analyzed by flow cytometry using the Attune^®^ NxT Acoustic Focusing Flow Cytometer (FACS).

### Effect of GA on Oxidative Burst of Bovine and Human Granulocytes

Granulocytes (5 × 10^5^ cells/ 250 μl) were stimulated with NCE, EP, and phorbol 12-myristate 13-acetate (PMA, 25 μM, Sigma-Aldrich) as a positive control. Immediately 2′,7′-dichlorofluorosceindiacetate (DCF, 10 μM) was added and all samples were incubated at 37°C and 5% CO_2_ for 30 min. Respective background controls were included in all assays. Mean green fluorescence intensity of all cells (X-Mean of BL-1) was recorded by FACS (see above) as relative measure of ROS production.

### Bacterial Strains

The following *S. aureus* strains were used: *S. aureus* USA300 (a community acquired methicillin-resistant strain), *S. aureus* Newman (a well-characterized laboratory strain), and *S. aureus* Rd5 [a multi-resistant clinical bovine mastitis isolate (23)]. The following *E. coli* strains were used: *E. coli* K1 (serotype O18:K1:H7; isolated from a child with meningitis, kindly provided by Dr. Gregor Zysk, Düsseldorf) (24) and *E. coli* (ATCC^®^ 25922™).

### Assay to Measure Uptake of Bioparticles

The assay was performed by pre-stimulation of bovine granulocytes (5 × 10^5^ cells/250 μl) with NCE at a concentration of 20 mg/ml for 30 min at 37°C. Unstimulated cells were used as a control for the phagocytic activity of granulocytes. Cytochalasin D (Applichem Panreac; 5 mg/ml) at a final concentration of 10 μg/ml was added to inhibit phagocytosis. Afterwards, 5 μl of *S. aureus* bioparticles (Sigma-Aldrich; 10 mg/ml) was added and incubated again for 30 min at 37°C. To stop the reaction, cells were put on ice and centrifuged at 266 × ġ for 10 min at 4°C. Afterwards, the pellet was washed twice with cold 1 × PBS to remove unbound bacteria and centrifuged at the same speed. Finally, cells were resuspended in 250 μl cold 1 × PBS and attached/intracellular bioparticles were analyzed by FACS (see above) using a BL2 laser.

### Gentamicin and Vancomycin Protection Assay

In this assay, frozen inocula of *S. aureus* strains and *E. coli* strains were used. They were prepared briefly as follows: bacteria were grown overnight in Brain Heart Infusion (BHI) (37°C for 18 h, shaking 200 rpm). The overnight culture was diluted 1:50 in fresh prewarmed BHI and grown until the exponential growth phase under the same growth conditions was reached. Inocula then were washed once with 1 × PBS or 0.9% NaCl and dissolved in 1 × PBS. Immediately afterwards, inocula were frozen in liquid nitrogen and stored at −80°C. The colony-forming units (CFU) of the frozen and re-thawed inoculum were determined by plating.

Bovine or human lithium heparinized blood (1 ml) was incubated for 2 h at 37°C under rotation (60 rpm) with NCE (dissolved in 0.9% NaCl, final concentration of 20 mg/ml NCE). An untreated sample (only 0.9% NaCl) was used as a control. Then, 2.5 × 10^7^ CFU of *S. aureus* or *E. coli* strains were added and incubated for 30 min at 37°C under rotation (60 rpm) to allow phagocytosis of bacteria. For *E. coli* 25922, 2.5 × 10^8^ CFU was added to bovine blood. Subsequently, samples were centrifuged at 1,500 × g for 5 min, the supernatant was removed, and the pellet was resuspended in 400 μl of 0.2% NaCl for 30 s to remove erythrocytes by hypotonic lysis. The suspension was adjusted to a physiological NaCl concentration by the addition of 1.6% NaCl and centrifuged. This step was repeated twice and finally the pellet was suspended in RPMI containing gentamicin (final concentration, 100 μg/ml; Sigma-Aldrich) to kill extracellular bacteria. For *S. aureus* RD5 (resistant to gentamicin), vancomycin was used at a final concentration of 5 μg/ml (Sigma-Aldrich). All samples were incubated for up to 4 h at 37°C under rotation (60 rpm). At 30, 60, 120, 180, and 240 min post-phagocytosis in the presence of antibiotic, 100 μl of the suspension was washed once with 0.9% NaCl. The cells were then lysed with distilled water, and intracellular bacteria were plated and quantified on sheep blood agar plates after overnight incubation for 18 h at 37°C. Viability of cells was tested with trypan blue after lysis of the erythrocytes.

### Formation of Neutrophil Extracellular Traps (NETs)

Bovine or human granulocytes (2 × 10^5^ cells/well) were seeded into 48-well-plates (Greiner bio; 100 μl/well) containing an 8-mm glass cover slip (Thermo Fisher Scientific Ø 8 #1) coated with Poly-L-Lysine (Sigma-Aldrich). NCE was added in different concentrations (1, 5, and 20 mg/ml). Methyl-β-cyclodextrin (CD; Sigma-Aldrich) was used as a NET inducer in bovine granulocytes (final concentration 10 mM) and phorbol 12-myristate 13-acetate (PMA) was used as a NET inducer in human granulocytes (final concentrations, 25 nM). As a negative control, only RPMI medium was added. The cells were then incubated at 37°C with 5% CO_2_ for either 90 min or 4 h. After incubation, the plates were centrifuged (370 ×g) for 5 min at room temperature and fixed with a final concentration of 4% paraformaldehyde (PFA) for 15 min at 20°C. The cells were stained as described previously (25) with mouse IgG2a anti-DNA/histone antibody (Millipore MAB3864; 0.55 mg; 1:1,000) or IgG2a from murine myeloma (Sigma-Aldrich; 0.2 mg/ml) as an isotype control in the presence of a blocking buffer. As secondary antibody, goat anti-mouse Alexa 633 (life technologies A21070) was added.

In human granulocytes, elastase was visualized with rabbit anti-elastase antibody (Millipore; 5.1 mg/ml; 1:300) and DNA/histone with mouse IgG2a anti-DNA/histone antibody (Millipore MAB3864; 0.55 mg; 1:1,000). As isotype controls, IgG2a from murine myeloma (Sigma-Aldrich; 0.2 mg/ml) or rabbit IgG (Jackson ImmunoResearch; ChromPure Rabbit IgG; 11.1 mg/ml) was used. As secondary antibody, goat anti-rabbit Alexa 633 (life technologies A21070) or goat anti-mouse Dylight488 (Thermo Scientific; 1:1,000) was added. Finally, human and bovine cells were stained with aqueous Hoechst 33342 (Sigma-Aldrich; 50 mg/ml; 1:1,000) in distilled water for 10 min and after washing embedded in ProLong Gold (Invitrogen).

NETs were visualized using a Leica TCS SP5 confocal inverted base fluorescence microscope with an HCX PL APO 40 × 0.75–1.25 oil immersion objective. Settings were adjusted with control preparations using an isotype control. Six randomly selected images were acquired per sample. NET formation was analyzed by counting NET-releasing cells and non-releasing cells using the ImageJ program (22). As NET-releasing cells, all cells were counted with DNA-histone off-shoot or showing at least two of the following criteria: (1) enlarged nucleus, (2) decondensed nucleus, or (3) blurry rim.

### Thin-Layer Chromatography (TLC)

TLC analysis of the different GA samples was performed according to the procedure described in European Pharmacopeia (4, 26) with some modifications. One milliliter of 5% NCE, CGA, and EP was hydrolyzed in 1 ml of trifluoroacetic acid (TFA, 2 M) for 4 h at 100°C. The samples were dried using a vacuum centrifuge and the dry residue was dissolved in 1 ml of distilled water. One-dimensional TLC analysis was performed with 20 × 10 cm silica gel plates (Merck). The solvent system used consisted of monosodium phosphate, 1-butanol, and acetone (10:40:50 vol/vol). Five percent standard mixture of galactose, rhamnose, and arabinose were analyzed in parallel as a standard. When the solvent front migrated 10 cm up the plate, the plate was air dried. Afterwards, the plate was incubated in the same running solution until 15 cm and dried in the oven for 10 min at 100°C. Sugars were detected by spraying the plate with hydrochloric acid, ethanol, and urea (5:20 100 g/vol/vol) and heated for 20 min. The quantification of monosaccharaide composition was performed by CP ATLAS analysis software and compared to standards run on the same TLC plate.

### Growth of *S. aureus* and *E. coli* in the Presence of GA and Artificial Sugar

Bacteria were grown on blood agar plates and in Mueller Hinton Broth (MHB) with Ca^2+^ 3.158 mg/L and Mg^2+^ 6.143 mg/L. For liquid cultures, one colony was incubated in MHB at 37°C for 18 h under continuous shaking (200 rpm). The overnight culture was diluted in the same fresh prewarmed medium and adjusted to an OD_600nm_ of 0.05. These cultures were added to NCE, CGA, EP, and artificial sugars. For this purpose, NCE, CGA, and EP were dissolved in MHB containing Ca^2+^ (3.158 mg/L) and Mg^2+^ (6.143 mg/L) and filtrated through a 0.45-μm pore size filter. Concentrations of GA preparations were used from 5 to 40 mg/ml. Artificial sugars rhamnose, arabinose, and galactose (RGA) were prepared in a ratio equivalent to the sugar content of NCE, which were quantified by TLC (14%:29%:36%). Bacteria and media were mixed in a 96-well-plate, followed by incubation in a TECAN 200 reader at 37°C. The growth was monitored by measuring the optical density OD_595nm_ for 14 h in 30-min intervals. All experiments were conducted in triplicate per each technical run and the mean was calculated. The data present the mean of three independent technical runs. In case of irregular results seen for a bacterial negative control, the whole experiment was excluded.

### Transmission and Scanning Electron Microscopy

Two hundred fifty microliters of *S. aureus* Newman (grown as described above in growth experiments) was added to 250 μl of NCE or EP (dissolved in MHB) with a final concentration of 40 or 5 mg/ml. As a negative control, only MHB was added. All samples were incubated for 8 h at 37°C and fixed with PFA (final concentration 4%). Each sample was gently transferred into a 1-ml sample tube and centrifuged at 2,500 × g for 20 min. Pellets were fixed in 2.5% (vol/vol) glutaraldehyde in 0.1 M sodium cacodylate (pH 7.2) for 2 h at 4°C and subsequently washed with 0.15 M cacodylate (pH 7.2). They were then post-fixed with 1% osmium tetroxide (wt/vol) and 0.15 M sodium cacodylate (pH 7.2) for 1 h at 4°C, washed, and further processed for electron microscopy.

For scanning electron microscopy, specimens were washed with cacodylate buffer and dehydrated with an ascending ethanol series from 50% (vol/vol) to absolute ethanol (10 min per step). As a positive control, LL-37 (final 3 mM) was used. The specimens were then subjected to critical point drying in carbon dioxide, with absolute ethanol as an intermediate solvent, mounted on aluminum holders, sputtered with 30 nm palladium/gold, and examined in a FEI Quanta FEG 3D scanning electron microscope.

For transmission electron microscopy, the fixed and washed samples were subsequently dehydrated in ethanol and further processed for standard Epon embedding. Sections were cut with an LKB ultratome and mounted on Formvar-coated copper grids. The sections were post-fixed with uranyl acetate and lead citrate and examined in a Philips/FEI CM100 BioTwin transmission electron microscope operated at a 60-kV accelerating voltage. Images were recorded with a Gatan Multiscan 791 charge-coupled device camera.

### Statistical Analysis

Data were analyzed by using Excel 2016 (Microsoft) and GraphPad Prism 8 (GraphPad Software). Each experiment was performed at least three times with blood from cows and humans, and within each experiment, all samples were processed at least in duplicate. Normal distribution of data was verified by Kolmogorov Smirnov normality test (GraphPad software) prior to statistical analysis. All statistical tests used are described in the respective figure legends. Based on the evidence that GA exhibits proinflammatory effects by complement activation (20), proinflammatory effects were hypothesized and a one-tailed test was used to quantify the *P*-value for the effects of GA on neutrophils. ^*^P < 0.05, ^**^P < 0.01, ^***^P < 0.001, and ^****^P < 0.0001 were considered significant.

## Results

### Time- and Concentration-Dependent Inhibition of Bacterial Growth by GA

Already in a previous study, the antimicrobial effect of five different crude extracts of Omani and Sudanese GA was investigated using a disc diffusion assay to determine antibacterial effects and also minimum inhibitory concentrations of various GA extracts against different Gram-positive as well as Gram-negative clinical pathogenic isolates such as *S. aureus* and *E. coli* strains. Concentrations of 2, 1, 0.5, and 0.25 mg/ml were tested by the authors for its antimicrobial activity and revealed bacteriostatic effects (27). To investigate in more detail to what extent GA has antibacterial effects, the growth of bacterial *S. aureus* and *E. coli* strains was monitored over time in the presence of GA in a concentration range in liquid cultures in our presented study.

**Figure 1:**
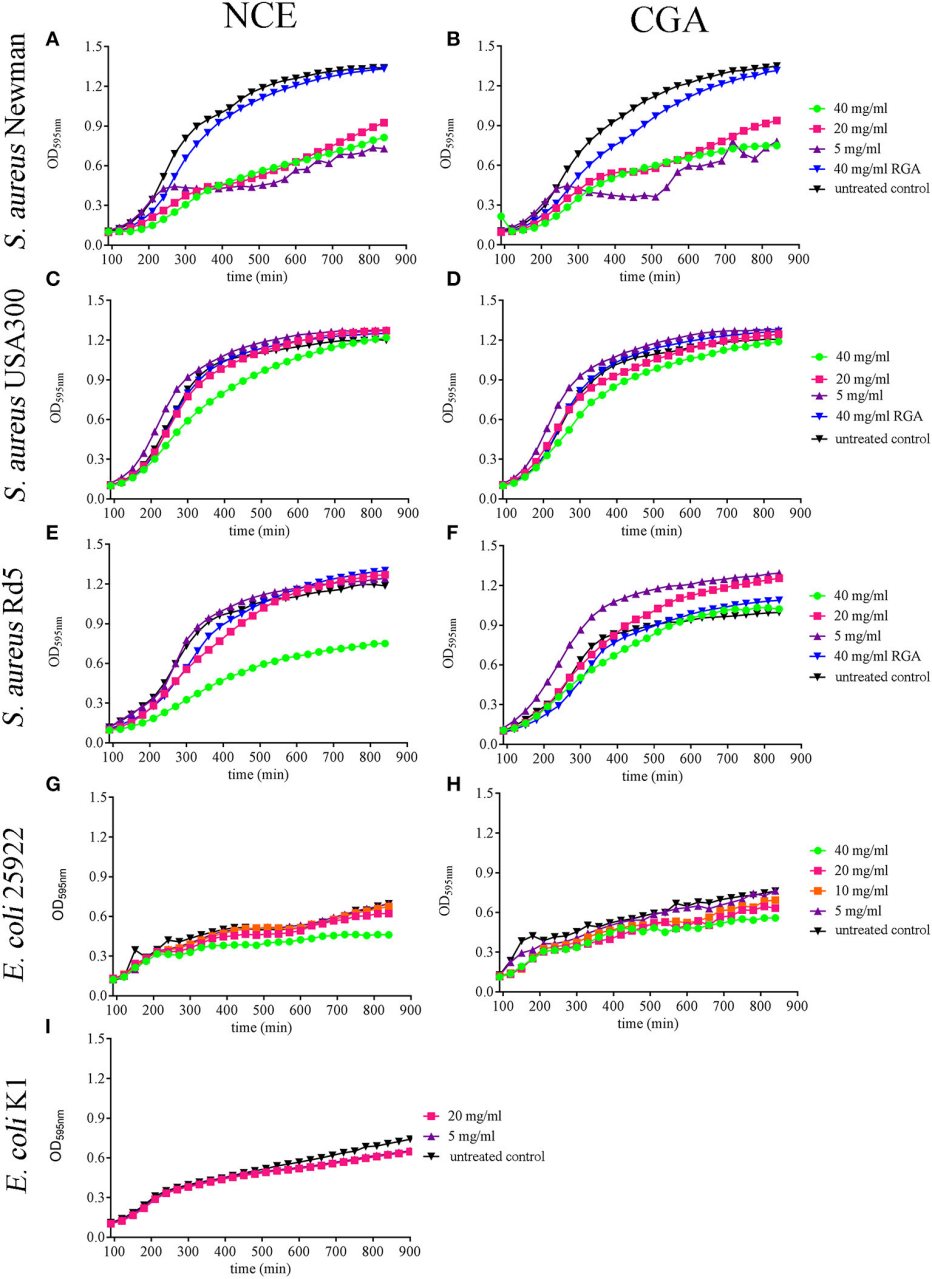
Effect of natural crude extract (NCE), commercial GA (CGA), and artificial sugars (RGA) on growth of *S. aureus* strains and *E. coli* strains in MHB over 14 h. The growth of different *S. aureus* and *E. coli* strains was analyzed in the presence of different GA concentrations by measuring the OD in a plate reader. **(A,B)** All tested concentrations of NCE **(A)** and CGA **(B)** significantly inhibited the growth of *S. aureus* Newman compared to the untreated control. The inhibition effect of 40 mg/ml NCE started at 180 min, 20 mg/ml at 210 min, and 5 mg/ml at 240 min. For 40 mg/ml and 20 mg/ml CGA, the growth-inhibiting effect started at 240 min and for 5 mg/ml at 300 min. Rhamnose, galactose, and arabinose (RGA = artificial sugars) had no effect on *S. aureus* Newman growth (*n* = 3). **(C,D)** Only 40 mg/ml of NCE **(C)** and CGA **(D)** slightly inhibited after 210 min the growth of *S. aureus* USA300 compared to the untreated control (NCE: *n* = 5 and CGA *n* = 6). This effect was lost over time. **(E,F)** Only 40 mg/ml NCE **(E)** significantly inhibited the growth of *S. aureus* Rd5 after 210 min, whereas CGA **(F)** had no effect (*n* = 3). **(G,H)** 40 mg/ml NCE and CGA significantly inhibited the growth of *E. coli* 25922. The effect started with NCE at 240 min **(G)** and with CGA at 480 min (*n* = 3). **(I)** NCE had no effect on the growth of *E. coli* K1 (*n* = 3). Statistical analysis was done by two-way ANOVA followed by Dunnett's test compared to the untreated control. Each treatment was done in duplicate and a mean of duplicates was used for statistical analysis. The number of independent experiments is presented above. *P* ≤ 0.05 were considered significant.

First, the growth of *S. aureus* Newman was significantly inhibited starting in a time- and concentration-dependent manner with 5 mg/ml NCE of GA (*P*_time_ < 0.0001; *P*_concentration_ < 0.0001; Figure 1A). The growth of *S. aureus* USA300 was slightly inhibited with 40 mg/ml NCE (*P*_time_ < 0.0001; *P*_concentration_ < 0.0001; Figure 1C). However, the growth-inhibiting effect was lost over time. In the case of *S. aureus* Rd5, a clearer significant time- and concentration-dependent inhibitory effect of GA was detected at 40 mg/ml NCE (*P*_time_ < 0.0001; *P*_concentration_= 0.0025; Figure 1E).

When testing the effect of GA on *E. coli* growth, *E. coli* 25922 was significantly inhibited after 4 h with 40 mg/ml NCE (*P*_time_ < 0.0001; *P*_concentration_ < 0.0001; Figure 1G). In the case of the tested *E. coli* K1 strain, a significant time-dependent growth inhibition was detectable for 5 and 20 mg/ml NCE after 11.5 h incubation (*P*_time_ < 0.0001; *P*_concentration_ = 0.2935; Figure 1I). For each strain and treatment, one selected time point from the growth curve is presented in Supplementary Figure 2 as a column diagram.

In summary, the strongest antibacterial effect was detectable with NCE on *S. aureus* Newman with concentrations starting from 5 mg/ml. Antibacterial effects were furthermore detectable with higher concentrations in other strains. Differences were less clear in the case of *E. coli*.

### Comparison of NCE, CGA, and EP on Bacterial Growth

To assess if the effect is specific for the NCE and if the sugar composition influences the activity of GA, we compared three types of GA: (1) CGA, (2) NCE from *Acacia Senegal* (L.) Willdenow trees from Sudan, and (3) EP generated from NCE.

As a control experiment, we first analyzed the purity and quantity of monosaccharides in GA by TLC (Supplementary Figure 3). The monosaccharide composition was analyzed in NCE and CGA samples and the results showed similar monosaccharide compositions. In addition, the EP was tested and showed significantly less monosaccharides (arabinose and galactose) compared to NCE as well as CGA as expected.

**Figure 2:**
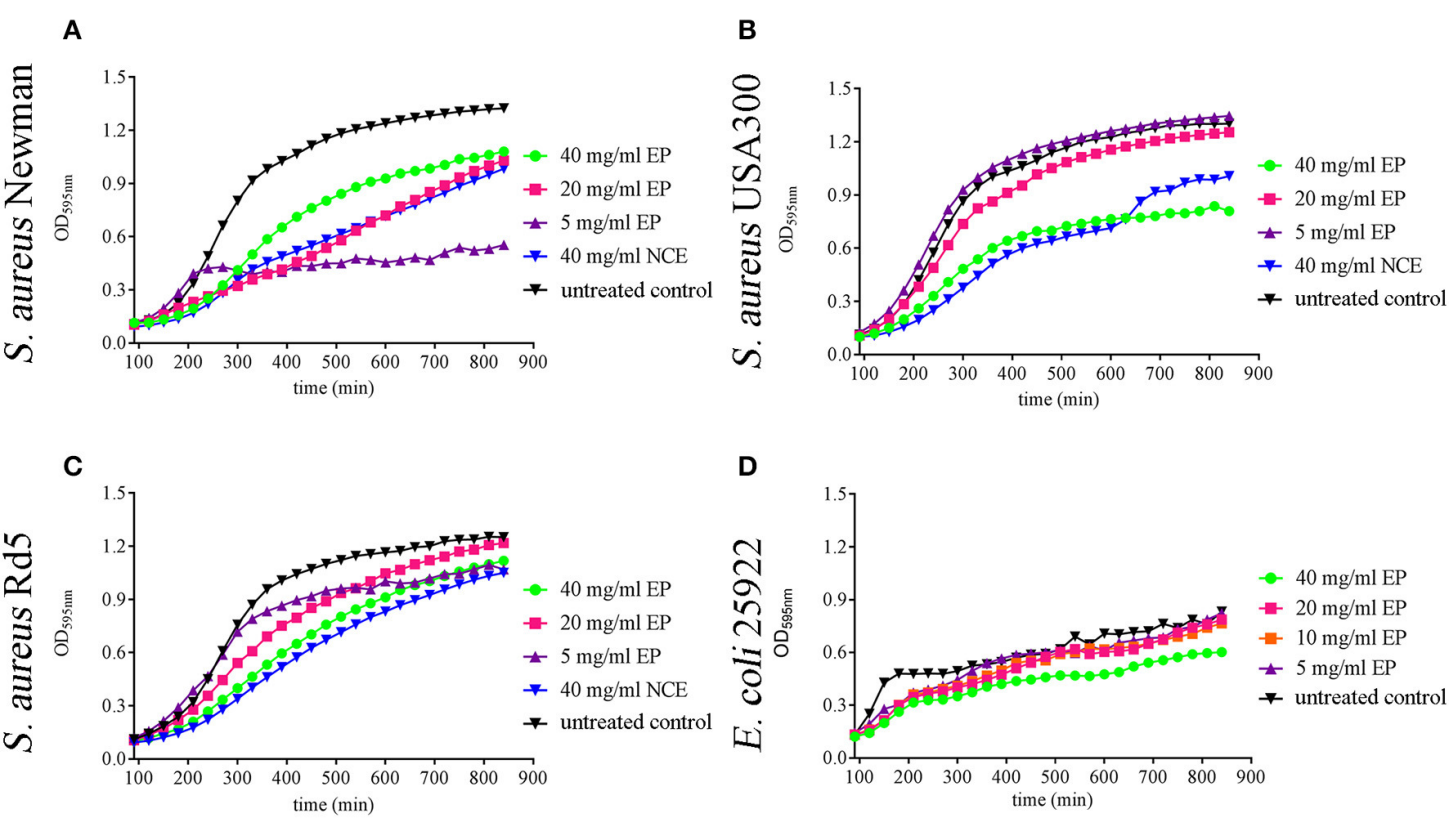
Effect of ethanol precipitate (EP) on growth of *S. aureus* strains and *E. coli* strains in MHB over 14 h. 20 mg/ml and 40 mg/ml EP **(A)** significantly inhibit the growth of *S. aureus* Newman at 240 min and 5 mg/ml inhibitory effect started at 270 min (*n* = 3). 40 mg/ml EP **(B)** significantly inhibits the growth of *S. aureus* USA300 at 210 min (*n* = 3). 40 mg/ml EP significantly inhibits the growth of *S. aureus* RD5 at 270 min **(C)** (*n* = 3). 40 mg/ml EP significantly inhibits the growth of *E. coli* 25922 at 120 min and the inhibitory effect of 20 mg/ml started at 150 min **(D)** (*n* = 3). Statistical analysis was done by two-way ANOVA followed by Dunnett's test compared to the untreated control. Each treatment was performed in duplicate and a mean value was used for statistical analysis. *P* ≤ 0.05 were considered significant.

Comparing NCE and CGA, a similar effect of GA was seen on growth inhibition of the tested bacteria (Figure 1). As the EP contains lower amounts of sugars compared to NCE (Supplementary Figure 3), we repeated the growth experiments with EP. Interestingly, EP also inhibited in a time- and concentration-dependent manner the growth of *S. aureus* Newman after 4 h even with 5 mg/ml EP (Figure 2A). The growth of *S. aureus* USA300 and Rd5 was only significantly inhibited with 40 mg/ml EP (Figures 2B,C). For *E. coli*, a significant time- and concentration-dependent growth inhibition was detectable for 40 mg/ml EP after 2.5 h and during the experiment even detectable at lower concentrations. However, the effect with lower concentrations was not stable until the end of the 14-h growth experiment (Figure 2D). For each strain and treatment, one selected time point from the growth curve is presented in Supplementary Figure 4 as a column diagram.

In summary, these data show that also the EP with a lower concentration of sugars exhibits an antibacterial effect. In good correlation with these data, the sugars alone showed no significant effect on bacterial growth (artificial sugars; Figure 1).

### Visualization of *S. aureus* After Treatment With NCE and EP Revealed Antibacterial Effects of GA

**Figure 3:**
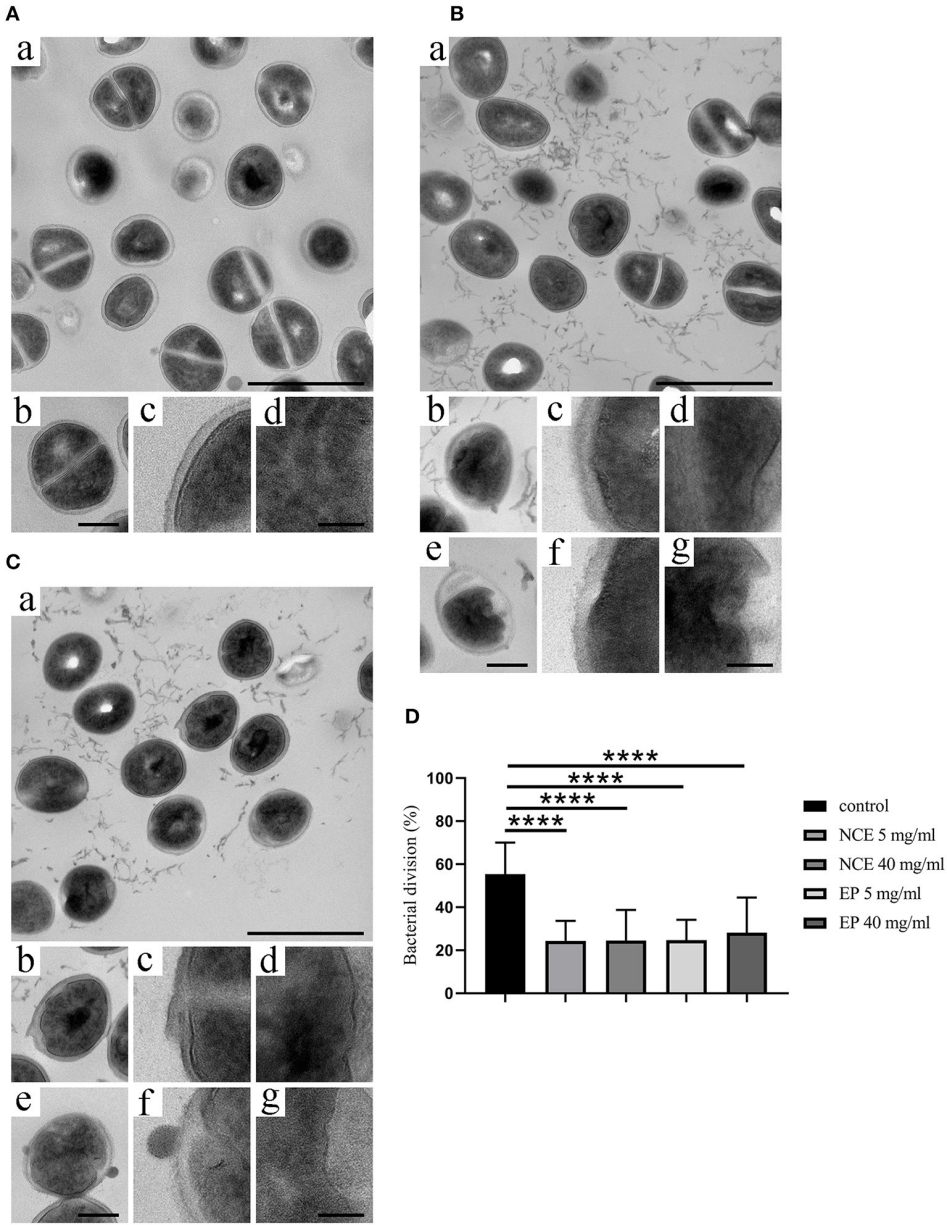
Natural crude extract (NCE) and ethanol precipitate (EP) inhibited cell division of *S. aureus. S. aureus* Newman was incubated for 8 h in the presence of **(A)** MHB medium as negative control, **(B)** NCE, and **(C)** EP. Samples were embedded in Epon, thin sectioned, and analyzed with transmission electron microscopic analysis. **(A)** Incubation with MHB showed no surface changes. (a) Overview; the scale bar represents 1 μm. (b) A representative bacterium at higher magnification, exhibiting an undisturbed architecture with homogenous cytoplasm and a central plane of cell division. The scale bar represents 250 nm. (c,d) Higher magnification of cellular aspects exhibiting the homogenous bacterial cell wall (c) and the homogenous cytoplasm (d). The scale bar represents 100 nm. **(B,C)** *S. aureus* Newman were analyzed with electron microscopy after incubation with 5 mg/ml and 40 mg/ml NCE **(B)** or 5 mg/ml and 40 mg/ml EP **(C)**. Incubation with 5 mg/ml NCE or EP (a–d), or 40 mg/ml NCE or EP (e,f), respectively. (a) Overview. NCE and EP generate an antibacterial effect with moderate morphological changes in bacterial architecture, occasional cytoplasmic blebbing, and less frequent central planes of cell division. The scale bar represents 1 μm. (b,e) Representative bacteria at higher magnification are shown, exhibiting a moderately disturbed architecture with less electron dense cytoplasmic regions and the absence of a central plane of division. The scale bar represents 250 nm. (c,d,f,g) Higher magnification of cellular aspects exhibiting the bacterial cell wall, which appears thicker and irregular as compared to the control (c,f). The cytoplasm appears heterogeneous and contains areas of less electron density (d,g). The scale bar represents 100 nm. **(D)** Percentage of the bacterial division was calculated. The percentage of dividing cells is lower in the NCE- and EP-treated group compared to growth control. Statistical analysis was done by one-way ANOVA followed by Dunnett's test compared to media control. Per treatment, 30 bacteria were counted in one viewing field and a total of 50 viewing fields were analyzed. All data are shown as means ± SD. ^****^P ≤ 0.0001 was considered significant.

**Figure 4:**
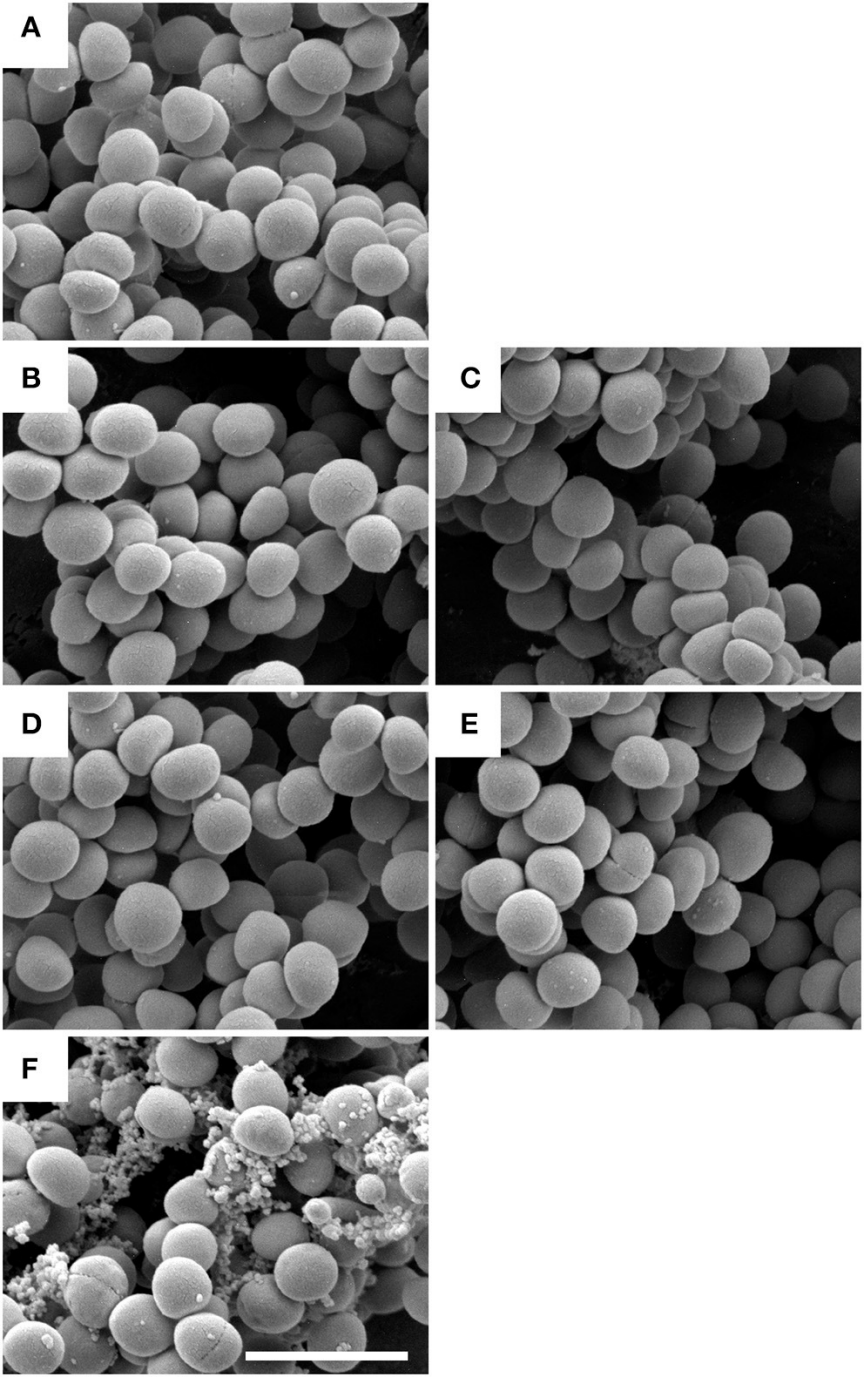
Scanning electron microscope (SEM) analysis showed no bactericidal effect by natural crude extract (NCE) and ethanol precipitate (EP) on *S. aureus* Newman. *S. aureus* Newman bacteria were incubated with 5 mg/ml NCE **(B)**, 40 mg/ml NCE **(C)**, 5 mg/ml EP **(D)**, or 40 mg/ml EP **(E)** respectively. They were compared to control bacteria without bactericidal treatment **(A)** and to bacteria treated with 3 mM LL-37 **(F)**. LL-37 induced membrane destabilization and cytoplasmic exudation, whereas the outer appearance of *S. aureus* bacteria appears largely undisturbed in **(A–E)**. The scale bar represents 2 μm.

To visualize the direct antibacterial effects of NCE and EP on *S. aureus* Newman, electron microscope techniques were used. By transmission electron microscopy, moderate morphological changes in bacterial architecture, occasional cytoplasmic blebbing, and less frequent central planes of cell division were seen after NCE and EP incubation of *S. aureus* Newman (Figures 3B,C). This effect was absent in bacteria grown without treatment (Figure 3A). The cell division was defined as a complete central plane in the cell as a well-established structural feature of bacterial division (28) and quantified by counting 30 bacteria in one viewing field. A total of 50 viewing fields were counted. In the untreated control, 55.3 ± 14.8% cells were dividing, whereas in NCE- and EP-treated samples, ~25% of the cells were dividing (Figure 3D). Background material found in the GA-treated samples is apparently related to structures present in the GA extract, as disrupted bacteria look structurally different (29). The density of bacteria was equal in all samples and no apparent cell loss was detectable. Therefore, the treatment resulted in highly significant differences in the percentage of dividing cells. Conversely, scanning electron microscopic analysis revealed neither membrane destabilization nor cytoplasmic exudation of *S. aureus* Newman after NCE or EP treatment (Figures 4B–E). Treatment of bacteria with the antimicrobial peptide LL-37, which is well-known for its cytotoxic effect on bacteria, was used as a positive control and revealed membrane destabilization and cytoplasmic exudation (Figure 4F). In summary, electron microscopy confirmed a bacteriostatic rather than a bactericidal effect on the bacteria.

Thus, after confirming the antibacterial effects of GA against *S. aureus* and *E. coli*, the immunomodulatory properties were characterized in detail in subsequent experiments.

### Effect of GA on the Oxidative Burst Activity in Human and Bovine Granulocytes

Peripheral blood granulocytes represent the first line of defense against bacterial infections by exhibiting intra- and extracellular antimicrobial activities, e.g., phagocytosis or formation of neutrophil extracellular traps, respectively. A key event in those mechanisms is the oxidative burst and formation of reactive oxygen species that initiate those processes (30). Thus, the next step in this study was to investigate the effect of GA extracts on the oxidative burst activity of granulocytes. Therefore, we isolated primary blood-derived granulocytes by density gradient centrifugation from fresh human or bovine blood. Firstly, as a control experiment, the cytotoxic effect of NCE on freshly isolated cells was tested with propidium iodide followed by FACS analysis. No cytotoxic effect on bovine granulocytes was detectable in a time frame until 120 min of incubation with up to 40 mg/ml NCE (Supplementary Figure 5). Furthermore, the concentration of LPS was tested with the LAL assay to exclude unspecific reactions by LPS contamination. The LPS amount in the NCE and EP was under the detection limit of the LPS assay and therefore <0.1 EU/ml and thus, less than the biologically active concentration (31).

**Figure 5:**
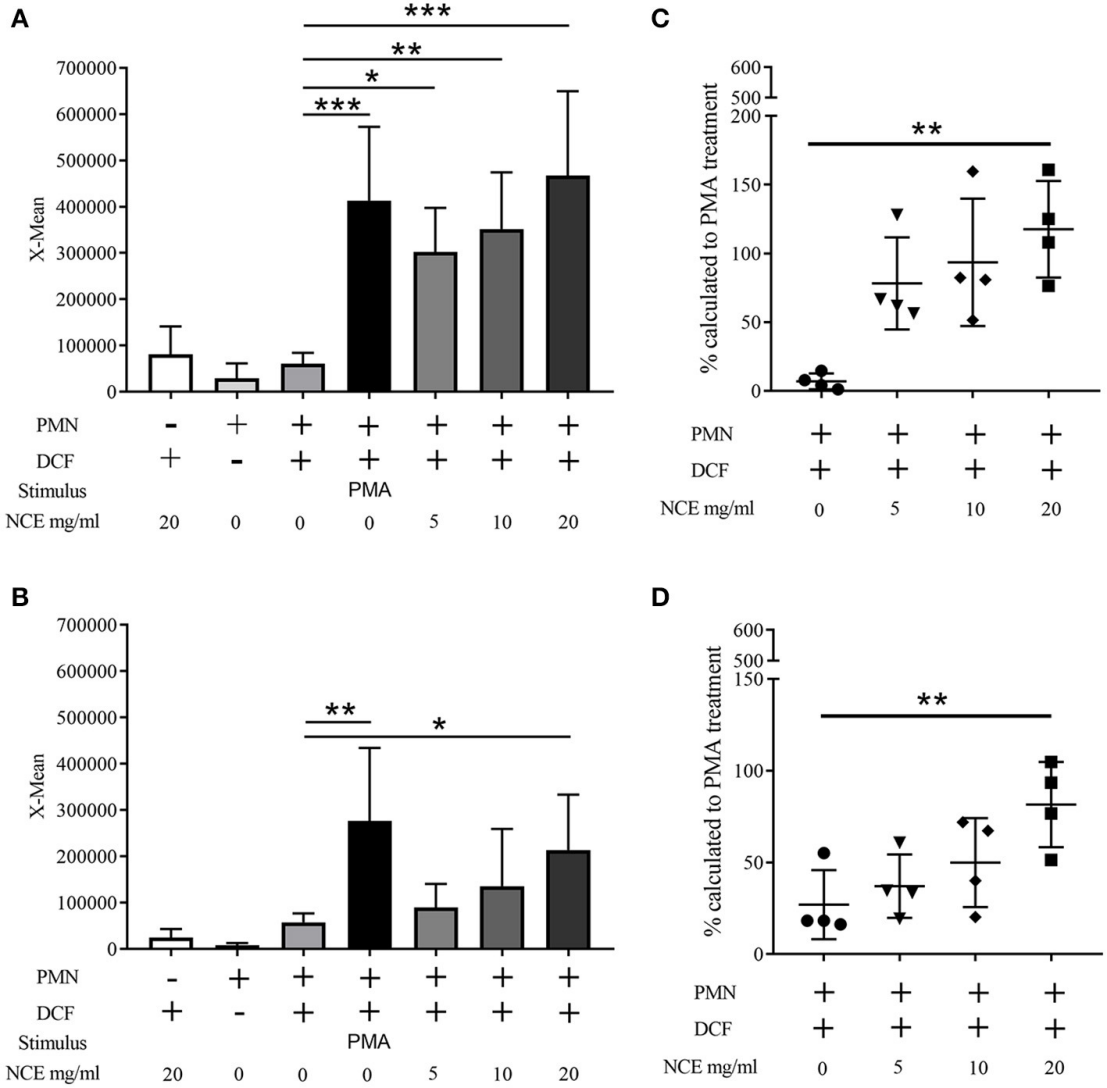
Effect of natural crude extract (NCE) on oxidative burst (ROS production) of human and bovine granulocytes. Mean fluorescence intensity (X-mean) of ROS was measured by FACS (BL-1) using DCF fluorescence probe (*n* = 4). Phorbol 12-myristate 13-acetate (PMA) was used as a positive control to induce ROS production. NCE significantly induces ROS production in human granulocytes **(A)** and bovine granulocytes **(B)** compared to unstimulated cells (cell + DCF). Additionally, the relative value to the PMA-stimulated cells was calculated and is presented as percentage **(C,D)**. Each dot represents one experiment. All data are shown as means ± SD. Statistical analysis was conducted with one-way ANOVA repeated measurement followed by Dunnett's multiple comparisons test **(A,B)** or in the case of non-normal distributed data by Friedman multiple comparisons test **(C,D)** to unstimulated cells. ^*^P < 0.05, ^**^P < 0.01, and ^***^P < 0.001 were considered significant.

Having confirmed that our extract does not exhibit a cytotoxic potential or harbors a significant amount of LPS contamination, the oxidative burst activity of human and bovine granulocytes was determined after 30 min treatment with 0–20 mg/ml NCE. Therefore, samples were analyzed by detecting the mean fluorescence intensity of DCF with a flow cytometer. The conversion of non-fluorescent dichlorofluorescin diacetate (DCFH-DA) to the highly fluorescent compound 2′,7′-dichlorofluorescein (DCF) can be used to monitor the oxidative burst in polymorphonuclear leukocytes. Background controls and unstimulated cells showed very low fluorescence signals (human unstimulated X-mean = 61,235 ± 23,361, bovine unstimulated X-mean = 57,573 ± 19,527), whereas the positive control treated with PMA showed significantly higher values (human *P* < 0.0001; bovine *P* = 0.0002). Interestingly, the human cells reacted stronger to PMA (X-mean = 413,481 ± 159,197) compared to bovine cells treated with PMA (X-mean = 276,753 ± 157,719). In human samples, NCE treatment of granulocytes significantly induced oxidative burst in a dose-dependent manner compared to unstimulated cells. After the stimulation with 20 mg/ml NCE, a comparable oxidative burst as detected after treatment with PMA was observed (X-mean = 467,278 ± 182,700) (Figure 5A). In bovine granulocytes, NCE also distinctly induced ROS activity in a dose-dependent manner, but in contrast to human cells, only 20 mg/ml showed a significant ROS induction (X-mean = 213,793 ± 119,965), and this value was still below the value reached after PMA stimulation (Figure 5B). It is obvious that the oxidative burst data are highly variable, especially for the human neutrophils; therefore, all data were additionally expressed relative to the positive PMA-induced response (Figures 5C,D). The relative value calculated in percentage to the PMA-treated cells showed in human as well as bovine neutrophils a significant higher ROS production after treatment with 20 mg/ml NCE compared to untreated cells (human *P* = 0.003; bovine *P* = 0.003; Figures 5C,D).

In summary, NCE of GA is able to induce an oxidative burst in human and bovine granulocytes.

### EP Induced ROS Production in Human and Bovine Granulocytes

**Figure 6:**
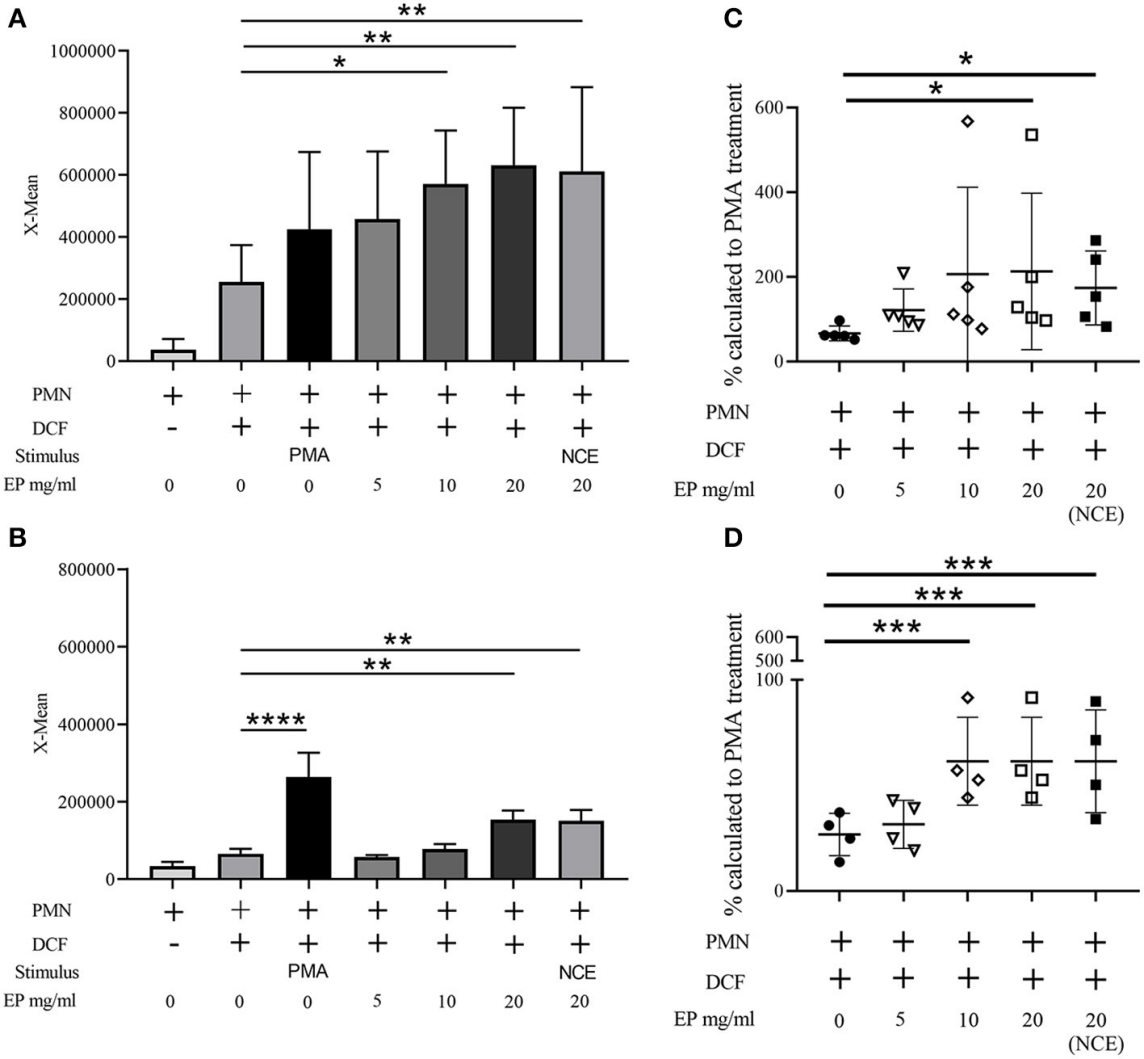
Effect of ethanol precipitate (EP) on oxidative burst (ROS production) of human and bovine granulocytes. Mean fluorescence intensity (X-mean) of ROS was measured by FACS (BL-1) using DCF fluorescence probe **(A)** *n* = 5; **(B)** *n* = 4. Phorbol 12-myristate 13-acetate (PMA) was used as a positive control to induce ROS. EP significantly induces ROS production in human granulocytes **(A)** and bovine granulocytes **(B)** compared to unstimulated cells (cell + DCF). Additionally, the relative value to the PMA stimulated cells was calculated and is presented as percentage **(C,D)**. Each dot represents one experiment. All data are shown as means ± SD. Statistical analysis was conducted with one-way ANOVA repeated measurement followed by Dunnett's multiple comparisons test **(A,B,D)** or in the case of non-normal distributed data by Friedman multiple comparisons test **(C)** to unstimulated cells. ^*^P < 0.05, ^**^P < 0.01, ^***^P < 0.001, and ^****^P < 0.0001 were considered significant.

Since EP, which contains fewer sugars, is able to exhibit antibacterial effects against *S. aureus*, similar to the NCE, we were interested if EP induces an oxidative burst in bovine and human granulocytes. Therefore, the oxidative burst activity of human and bovine granulocytes was determined after 30 min treatment with 0–20 mg/ml EP similarly as shown in Figure 1. As seen for NCE, EP significantly increased intracellular ROS production in a dose-dependent manner after treatment of human as well as bovine granulocytes (Figure 6).

### Phagocytosis of *E. coli* and *S. aureus* by Blood-Derived Granulocytes After NCE Stimulation

As oxidative burst is involved in phagocytosis, we were interested if GA-treated cells exhibit an altered phagocytic and intracellular killing activity. First, we analyzed if bovine granulocytes phagocytose bioparticles more efficiently after NCE treatment. Freshly isolated primary bovine granulocytes were treated for 30 min with 0, 5, and 20 mg/ml NCE. Afterwards, the stimulated cells were co-incubated with *S. aureus* fluorescence-labeled bioparticles. Then, cells were analyzed by flow cytometry and cells with ingested/associated bioparticles were detected. The uptake/association of bioparticles by granulocytes was significantly higher after stimulation with 5 mg/ml NCE compared to untreated cells (Supplementary Figure 6) but did not reach higher values with a higher concentration of NCE. As a control experiment, association/uptake was significantly reduced by adding cytochalasin D to block the cytoskeletal rearrangement, which is necessary for phagocytosis.

**Figure 7:**
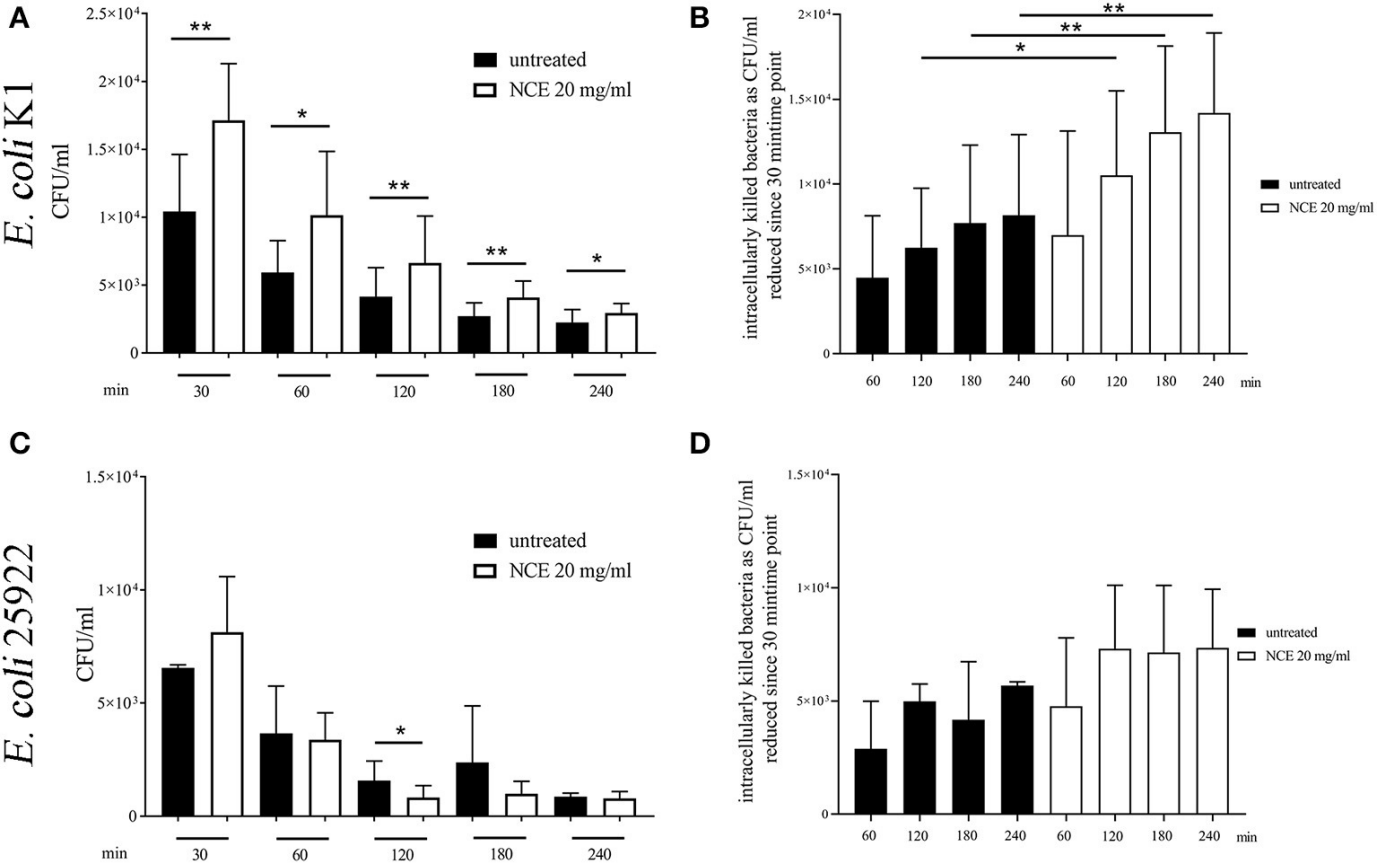
Phagocytosis of *E. coli* strains after pre-simulation of bovine and human whole blood with 20 mg/ml natural crude extract (NCE). Human and bovine whole blood were pre-stimulated for 2 h with 20 mg/ml NCE. Afterwards, human blood was co-incubated with *E. coli* K1 **(A,B)** and bovine blood with *E. coli* 25922 **(C,D)** for 30 min. Extracellular bacteria were killed with gentamicin. Untreated blood was used as a control. Phagocytosed *E. coli* K1 were recovered by lysing the eukaryotic cells and plating the samples on blood agar. Cells of NCE-stimulated human blood phagocytosed significantly more *E. coli* K1 after 30 min of co-incubation compared to the control **(A)**, and intracellular killing of *E. coli* K1 was higher in the NCE-treated group compared to the control **(B)**. Cells of NCE stimulated bovine blood had a tendency to phagocytose more *E. coli* 25922 after 30 min **(C)**. The number of intracellular killed *E. coli* 25922 was slightly increased in NCE-treated cells **(D)** compared to untreated cells. The assay was performed with blood from healthy donors (human *n* = 5, bovine *n* = 3) as independent experiments. All data are shown as means ± SD. Statistical analysis was done by one-tailed, paired Student's *t*-test. ^*^P < 0.05 and ^**^P < 0.01 were considered significant.

As living bacteria influence the phagocytosis more than inactive bioparticles, we analyzed in the next step the influence of GA treatment on living *E. coli*, as the bacterium is typically killed intracellularly. A gentamicin-protection assay was conducted with 2 h NCE pre-stimulation of whole blood and afterwards the addition of bacteria for up to 240 min. The phagocytosis rate (30 min) and killing rate (30, 60, 120, 180, and 240 min) were compared to an untreated group. As the ROS production was higher in human granulocytes, we conducted the assay with human blood and the human isolate *E. coli* K1. As a control experiment, the viability of the blood cells was tested and revealed no difference during the assay (Supplementary Figure 7). In good correlation with the increased ROS-production after GA treatment of granulocytes, also the phagocytosis rate of viable *E. coli* by blood-derived immune cells was significantly higher after treatment with NCE (*P*_30*min*_ = 0.0037; Figure 7A). Furthermore, the killing of intracellular bacteria was also significantly higher in the NCE-treated cells over time (Figure 7B). Similar experiments were performed with the laboratory strain *E. coli* ATCC 25922 in whole bovine blood. The NCE-treated blood showed not a significant, but a remarkably higher phagocytosis rate after 30 min of co-incubation with *E. coli* (Figure 7C) and a remarkably faster and therefore more efficient killing rate (Figure 7D). Thus, a similar trend is seen with another strain and bovine granulocytes; however, the NCE-dependent effect might differ slightly between different *E. coli* strains and/or blood from different animal species.

**Figure 8:**
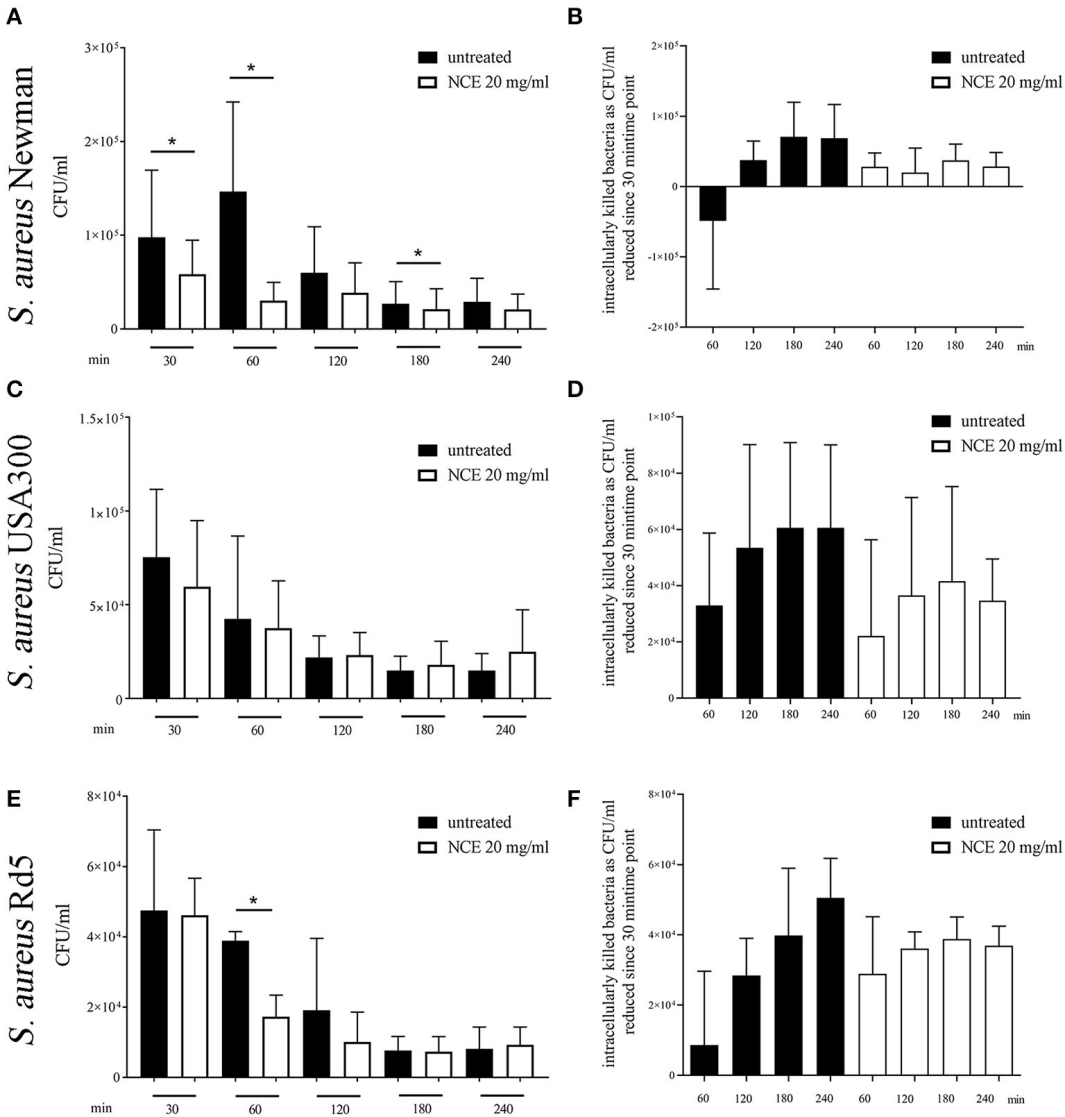
Phagocytosis of *S. aureus* strains after pre-simulation of bovine and human whole blood with 20 mg/ml natural crude extract (NCE). Human or bovine whole blood was pre-treated for 2 h with 20 mg/ml NCE. Human blood was co-incubated with *S. aureus* Newman (**A,B**; *n* = 5) and *S. aureus* USA300 (**C,D**; *n* = 5). Bovine blood was co-incubated with *S. aureus* Rd5 (**E,F**; *n* = 3). After 30 min of co-incubation, extracellular bacteria were killed with gentamicin **(A–D)** or vancomycin **(E,F)**. Untreated blood was used as a control. Phagocytosed *S. aureus* were recovered by plating the samples on blood agar after lysis of the eukaryotic cells. Cells of NCE-stimulated human blood phagocytosed significantly less *S. aureus* Newman after 30 min **(A)** and the killing of *S. aureus* Newman was comparable in untreated and NCE-treated blood **(B)**. Phagocytosis of *S. aureus* USA300 was not significantly different in untreated and treated human blood **(C,D)**. NCE stimulation of bovine blood had no effect on the uptake of *S. aureus* Rd5 **(E)**, but the killing of intracellular bacteria is remarkably lower after 30 min **(F)**. All data are shown as means ± SD. Statistical analysis was done by one-tailed, paired Student's *t*-test. ^*^P < 0.05 was considered significant.

In the next step, we investigated if the phagocytosis and killing rate of a mainly extracellularly killed bacterium is influenced by NCE treatment. Therefore, the assay was conducted with human blood and an MSSA (methicillin-sensible *S. aureus*, Newman) and an MRSA (methicillin*-*resistant *S. aureus*, USA300) strain. Furthermore, the assay was conducted with bovine blood and a methicillin-resistant *S. aureus* strain (Rd5, bovine mastitis isolate). In contrast to *E. coli*, the phagocytosis rate of *S. aureus* Newman was significantly reduced in the NCE-treated group (*P*_30*min*_ = 0.0418; Figure 8A). Interestingly, there was a tendency that overall less bacteria were found intracellularly in case of all *S. aureus* strains (Figure 8A) and at the same time less bacteria were killed intracellularly (Figure 8B).

Summarizing this part, NCE treatment increased the phagocytosis and killing rate of the intracellularly killed bacterium *E. coli* in human blood. A similar tendency was seen in the case of bovine blood with an *E. coli* strain. The NCE treatment did not increase the phagocytosis rate of the predominately extracellular pathogen *S. aureus* in bovine or human blood.

### NCE Did Not Induce Neutrophil Extracellular Traps (NETs)

**Figure 9:**
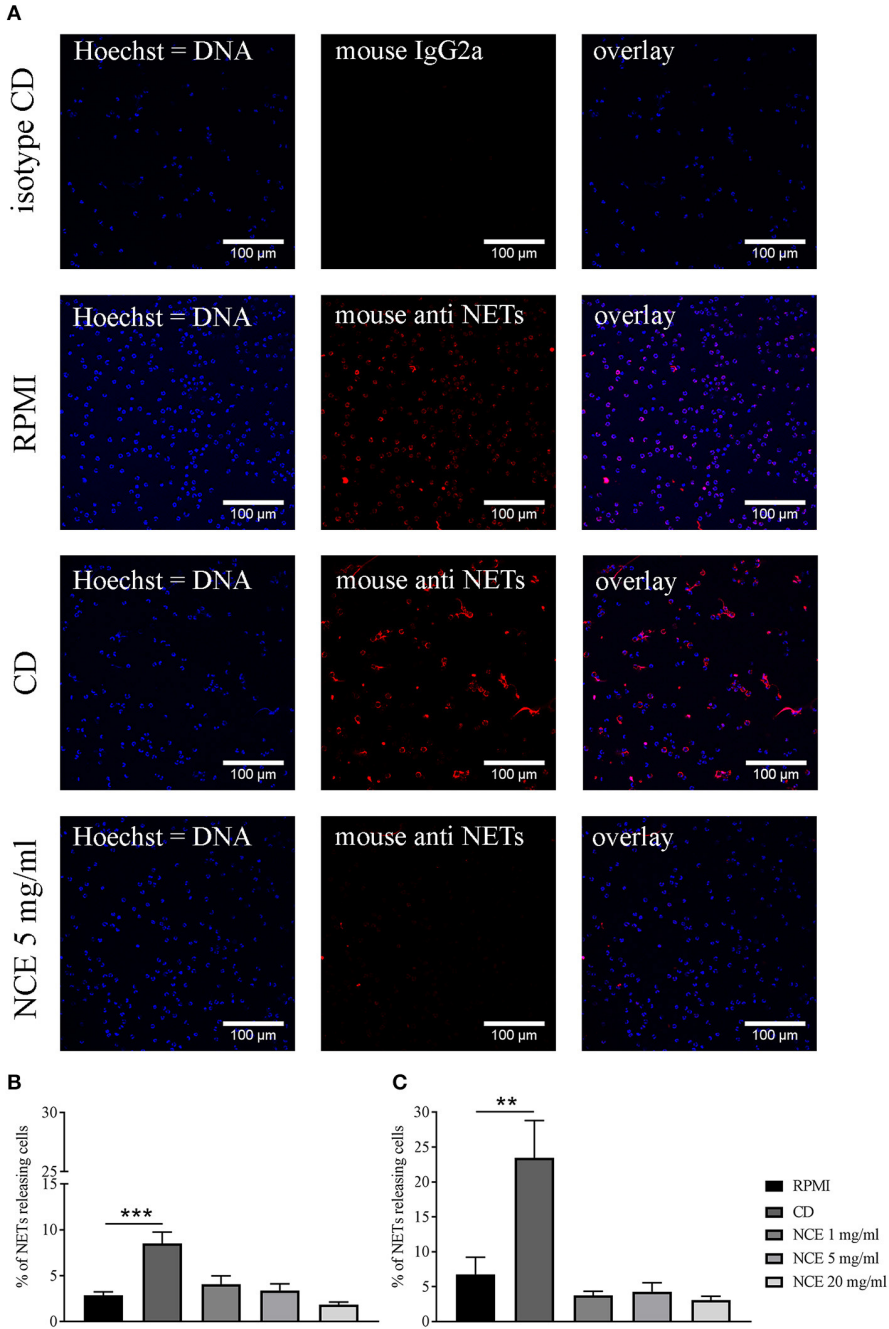
Formation of bovine neutrophil extracellular traps (NETs) after 90 min and 4 h stimulation with natural crude extract (NCE). Bovine neutrophils were stimulated with methyl-β-cyclodextrin (CD) as a positive NET inducer, RPMI medium as a negative control, and different concentrations of NCE. NETs were visualized with immunofluorescence microscopy after adjustment to an isotype control. Representative pictures are presented in **(A)**. NCE-treated samples showed no NET induction **(A)**. Images were quantified using ImageJ program. **(B)** Samples were incubated for 90 min. **(C)** Samples were incubated for 4 h. There was no significant difference between RPMI-treated groups and NCE-treated groups independent of concentration and time (*n* = 5). A total of six randomly selected images were taken in each experiment for each sample. All data are shown as means ± SD. Statistical analysis was done using one-way ANOVA repeated measurement comparing to RPMI control followed by Dunnett's multiple comparisons test. ^**^P < 0.01 and ^***^P < 0.001 were considered significant.

Besides phagocytosis, ROS is also involved in NET formation, which is one important extracellular mechanism of granulocytes to counteract bacterial infections, especially against extracellular pathogens, e.g., *S. aureus* (32, 33). Therefore, the ability of NCE to induce NETs as an innate immune defense mechanism was evaluated in a concentration- and time-dependent manner. Since during NET formation often unspecific reactions of granulocytes can be seen by treating cells with the vehicle controls, lower concentrations of 1 mg/ml GA were included here to exclude side effects by vehicle control. Finally, GA had no effect on NET formation at all, also not with higher concentrations of GA: As seen in Figure 9, no significant differences between NCE-treated groups and the negative controls were detectable over time (90 min and 4 h) in bovine granulocytes (Figure 9) or human granulocytes (Supplementary Figure 8). The NCE treatment did not lead to an increased extracellular immune cell–pathogen interaction by NET formation.

Summarizing all data, NCE of GA significantly increased the oxidative burst of bovine and human granulocytes and may enable a more efficient killing of intracellular pathogens, e.g., *E. coli*. GA was not shown to enhance the formation of extracellular traps, which may act against extracellular pathogens such as *S. aureus*. However, GA was able to inhibit the growth of *S. aureus*, an effect that was not mediated by the sugars alone. EP showed a similar effect, confirming that the effects of GA are not mediated by the sugars alone but by the complex composition including additional components of the GA extracts.

## Discussion

Antibiotic therapy helps the host to reduce bacterial infections, but multi-resistant bacteria are becoming a major health problem in the twenty-first century (34). The pathogenic bacterium *S. aureus*, for example, can rapidly adapt to the selective pressure of antibiotics and this resulted in the emergence and spread of methicillin-resistant *S. aureus* (MRSA) during the recent years (35). Identification of natural products with direct antimicrobial effects or also immunomodulatory effects to boost the innate immune defense is a promising strategy to identify and characterize new treatment or prevention strategies (36). GA is a well-known traditional herbal medication from *Acacia Senegal* (L.) Willdenow trees, which for a long time has been used for several diseases but also as a treatment to protect against bacterial infections. We here investigated the immunomodulatory effect of GA on blood-derived granulocytes and the direct effect of GA on the pathogenic bacteria *S. aureus* and *E. coli*. For studying the immunomodulatory effect, pure granulocytes were used to see the direct effect of GA on the cells. In addition, a whole blood assay was used to see the effect of granulocytes in a more *in vivo* close reaction with all components including effect of plasma. As GA we used NCE and EP prepared from plant material collected in Sudan with a controlled quality and purity: (1) the detected LPS content was below the biologically active amount that may interfere with the immune cell function assays in this study. (2) None of the used concentrations exhibited cytotoxic effects against the granulocytes (Supplementary Figures 5, 7). (3) The sugar content in NCE reflects the described amount in the literature (2), tested with methods described in the European Pharmacopeia (Supplementary Figure 3). As the growing area of the acacia trees may influence the GA composition (37), it was of interest if the sugar content is similar to a commercially available GA. Thus, we compared the sugar composition of the NCE with GA available from Sigma and revealed no significant differences (Supplementary Figure 3).

Although anti-inflammatory effects of GA have been described (7, 19), GA significantly increased oxidative burst/ROS production of bovine and human granulocytes in a dose-dependent manner (Figures 5, 6). Similarly, arabinogalactan–protein (AGP) of GA may have proinflammatory properties on certain parts of the immune system, e.g., the complement system. Lei et al. demonstrated an increased NO and ROS production of murine macrophages in a dose-dependent manner after 20 h incubation with a crude polysaccharide-enriched fraction of *Sutherlandia frutescens* (38). Moreover, certain types of *S. frutescens* polysaccharides extracted from leaves influence the complement system (39). ROS production leads to cell activation and can enhance the phagocytosis and killing of invading bacteria by phagocytes. Several evidences are shown in the literature that there is a direct link between increased ROS production and phagocytosis and the NADPH oxidase is a key enzyme in this process (40, 41). However, the heterogeneous nature and potential function of phagocytic vacuoles in various cells brings a high complexity into those processes. Furthermore, ROS is part of the oxygen-dependent NET formation [reviewed by (42, 43)]. NET formation acts as an extracellular mechanism of neutrophils against invading bacteria and can bind, disarm, and even kill extracellular bacteria such as *S. aureus*. NETs are described in many animal species, like human, cattle, pig, cat, fish, opossum, dog, and mouse (21, 44–51) and can be induced by bacteria, cytokines, and several other stimuli. However, GA does not induce NETs in human or bovine granulocytes (Figure 9 and Supplementary Figure 5), and therefore, it is unlikely that GA induces an extracellular NET-depending killing of bacteria.

Thus, we investigated in more detail if GA may influence the phagocytosis of typically intracellularly killed bacteria, e.g., *E. coli*. *E. coli* infections can lead to numerous diseases in different species, for example, life-threatening meningitis in humans and mastitis in cows. Therefore, in our study, we used a clinical isolate originally derived from a human meningitis patient. In good correlation to the GA-mediated increase in ROS production, GA treatment of whole human blood resulted in enhanced uptake and intracellular killing of the pathogenic *E. coli* K1 meningitis strain (Figures 7A,B). A better intracellular killing was also partially seen for the laboratory strain with bovine blood. However, in good correlation to the fact that *S. aureus* is mainly killed by extracellular pathways, e.g., NET formation, there was no clear effect of GA on intracellular killing by blood-derived granulocytes (Figure 8). Furthermore, it might also be speculated that enhanced phagocytosis of *E. coli* is not a general effect of GA on neutrophil function but also a combination of several effects in the whole blood environment including the described complement effects (20) that are only evident in the presence of *E. coli*.

It is important to mention that despite the fact that treatment of granulocytes with GA leads to increased uptake of *S. aureus* bioparticles (Supplementary Figure 6), fewer bacteria are found in granulocytes treated with GA at early time points during the intracellular killing assay using fresh blood (Figure 8). This phenomenon might be caused by a direct antimicrobial effect of GA on the bacteria. In this regard, it is especially interesting that GA significantly inhibited the growth of *S. aureus* and acted antibacterially in a dose- and time-dependent manner when simply testing the effect of GA extracts on bacterial growth (Figures 1–4). No clear effect, however, was seen in the case of *E. coli*.

The different cell wall construction of Gram-negative (e.g., *E. coli*) and Gram-positive bacteria (e.g., *S. aureus*) influence the susceptibility against different antibiotics and the response of immune cells via different toll-like receptors (52). Therefore, a different effect of GA on Gram-positive *S. aureus* compared to Gram-negative *E. coli* is conceivable. Indeed, the results of the gentamicin and vancomycin protection assays in whole blood look very different between *E. coli* and *S. aureus* (Figures 7, 8). Whereas, NCE enhanced the phagocytosis rate of *E. coli*, the phagocytosis rate of *S. aureus* was reduced after NCE treatment. An explanation is the direct antibacterial effect of NCE on *S. aureus* (Figure 1), especially in the case of *S. aureus* Newman where the growth is inhibited the most. With this strain, a significantly lower phagocytosis rate was detected (Figure 8A). Therefore, growth inhibition of bacteria by NCE treatment (Figure 3) may explain the lower phagocytosis rate. No clear effect of GA on phagocytosis was seen for *S. aureus* USA300 or Rd5, as in these strains, the direct bactericidal effect by NCE was also less pronounced (Figures 1, 8). Varying effects in different bacterial strains might also differ depending on the virulence factors expressed by the different isolates; e.g., *S. aureus* is able to counteract phagocytosis [reviewed by (53)] via the production of superoxide dismutase or also by different toxins.

The following *S. aureus* strains were used in our study: *S. aureus* USA300 (a community acquired methicillin-resistant strain), *S. aureus* Newman (a well-characterized laboratory strain), and *S. aureus* Rd5 [a multi-resistant clinical bovine mastitis isolate (23)]. USA300 is highly virulent, leading to overwhelming and tissue-destructive infections, such as necrotizing fasciitis and fulminant, necrotizing pneumonia based on toxin production, e.g., Panton-Valentine leukocidin (PVL) (54–56). *S. aureus* Newman is an antibiotic-susceptible strain isolated in 1952 from a human infection (secondary infection of a tuberculosis patient) (57). The strain displays robust virulence properties in animal models of disease and has already been extensively analyzed for its molecular traits of staphylococcal pathogenesis. But in contrast to the other strains, it harbors a natural mutation in the fibronectin binding protein A. In contrast, *S. aureus* Rd5 as bovine isolate is also an MRS strain, but this one is PVL negative and also negative for staphylococcal enterotoxins. These differences might explain the strain-dependent results, as the described virulence patterns might impact granulocyte behavior to varying effects.

In this study, we identified an immunomodulating effect of GA from *Acacia Senegal* (L.) Willdenow trees on granulocytes while concurrently exhibiting a direct antibacterial effect on pathogenic *S. aureus* isolates. Importantly, ROS production by GA as well as a bactericidal effect against *S. aureus* was not only detectable with NCE, but also with EP. EP in contrast to NCE contains less sugar. Therefore, we assume that the sugars alone are not the granulocyte-modulating or bactericidal factor in GA (Figure 6). This correlates with the fact that *E. coli* and *S. aureus* can use sugars like galactose for their own growth benefit (58, 59). Future work should focus on identifying the active compound or specific part of GA fractions with the described biological activity.

Furthermore, to test oral GA treatment possibilities, the indirect and direct effect on bacterial growth of GA, digested by intestinal bacteria, should be analyzed. Possibly active compounds of GA are released in the intestine and could explain results found during *in vivo* experiments, where GA-treated mice survived significantly longer after malaria infection (15). In good correlation with our findings regarding the antibacterial effect of GA, another study demonstrated direct antimicrobial effects of two different plant components of *Acacia* against various bacteria (60). In addition, extracts of *Acacia aroma* demonstrated antibacterial properties against methicillin-resistant and methicillin-sensitive staphylococci (61). Thus, identifying the active compounds in those *Acacia* species might help to characterize new treatment and/or prophylactic strategies against antibiotic-resistant bacteria.

## Data Availability Statement

All datasets generated for this study are included in the article/Supplementary Material.

## Ethics Statement

Human blood samples were collected from healthy donors with the help of human physicians in agreement with the local ethical board. The study was approved by the Ethic Committee of Hannover Medical School (MHH), Hannover, Germany, and registered under no. 3295–2016 and by the Ethic Committee of the University Medical Center Göttingen; Göttingen, Germany, under no. 3/5/15. The blood collection followed the local guidelines. The collection of blood from healthy female cows in the Clinic for cattle (University of Veterinary Medicine Hannover) was registered at the Lower Saxonian State Office for Consumer Protection and Food Safety (Niedersächsisches Landesamt für Verbraucherschutz und Lebensmittelsicherheit, Nos. 12A243 and 18A302). It was conducted in line with the recommendations of the German Society for Laboratory Animal Science (Gesellschaft für Versuchstierkunde) and the German Veterinary Association for the Protection of Animals (Tierärztliche Vereinigung für Tierschutz e. V.) (http://www.gv-solas.de).

## Author Contributions

NB, JS, HM, RN, MS, MK, and MK-B conceived and designed the experiments. SB, JS, TH, CF, MM, GS, and GB performed the experiments. NB, JS, MM, GB, and SB analyzed the data. SB, NB, and MK-B wrote the manuscript. All authors contributed to manuscript revision, and read and approved the submitted version.

### Conflict of Interest

MM was employed by the company Colzyx AB, Medicon Village, Lund, Sweden. MS was employed by the company LIONEX Diagnostics and Therapeutics, GmbH, Braunschweig, Germany. The remaining authors declare that the research was conducted in the absence of any commercial or financial relationships that could be construed as a potential conflict of interest.
