# Supplementary material for: Antimicrobial and Immunomodulatory Effect of Gum Arabic on Human and Bovine Granulocytes Against Staphylococcus aureus and Escherichia coli
Source: Front Immunol. 2020 Jan 31;10:3119. doi: 10.3389/fimmu.2019.03119 (PMC7005937; doi:10.3389/fimmu.2019.03119)
Supplement: Supplementary file 1 [file Data_Sheet_1.pdf]

Supplementary Material

Supplementary Figures

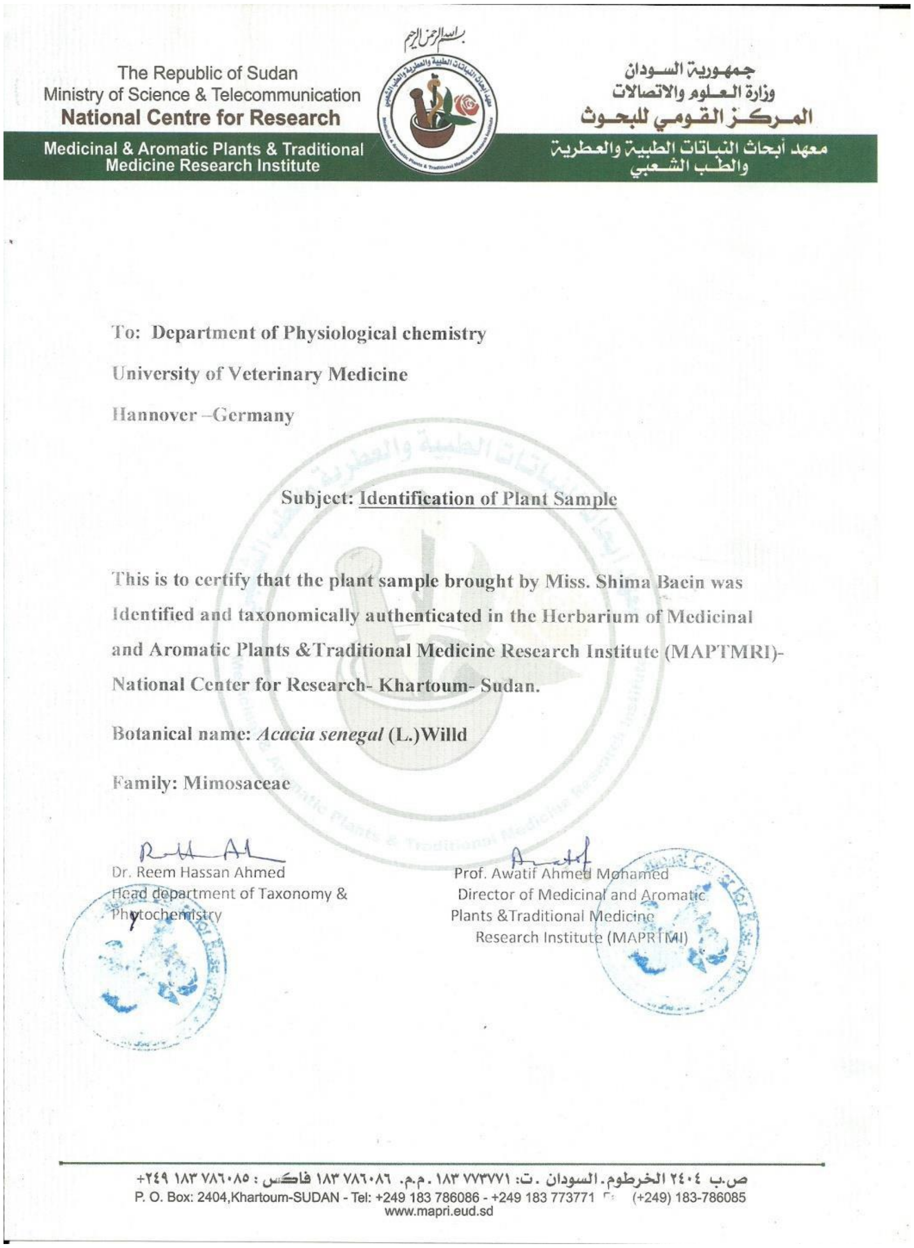

بسم الله الرحمن الرحيم

The Republic of Sudan
Ministry of Science & Telecommunication
National Centre for Research
Medicinal & Aromatic Plants & Traditional Medicine Research Institute

جمهورية السودان
وزارة العلوم والاتصالات
المركز القومي للبحوث
معهد أبحاث النباتات الطبية والعطرية والطب الشعبي

To: Department of Physiological chemistry
University of Veterinary Medicine
Hannover –Germany

Subject: <u>Identification of Plant Sample</u>

This is to certify that the plant sample brought by Miss. Shima Baein was Identified and taxonomically authenticated in the Herbarium of Medicinal and Aromatic Plants &Traditional Medicine Research Institute (MAPTMRI)- National Center for Research- Khartoum- Sudan.

Botanical name: ***Acacia senegal*** **(L.)Willd**

Family: Mimosaceae

Dr. Reem Hassan Ahmed
Head department of Taxonomy & Phytochemistry

Prof. Awatif Ahmed Mohamed
Director of Medicinal and Aromatic Plants &Traditional Medicine Research Institute (MAPRTMI)

ص.ب ٢٤٠٤ الخرطوم. السودان . ت: ١٨٣ ٧٧٣٧٧١ م.م. ١٨٣ ٧٨٦٠٨٦ فاكس : ١٨٣ ٧٨٦٠٨٥ ٢٤٩+
P. O. Box: 2404,Khartoum-SUDAN - Tel: +249 183 786086 - +249 183 773771 F: (+249) 183-786085
www.mapri.eud.sd

Supplementary Figure 1. Identification certificate to approve that Gum Arabic was collected from *Acacia Senegal* (L.) Willdenow tree.

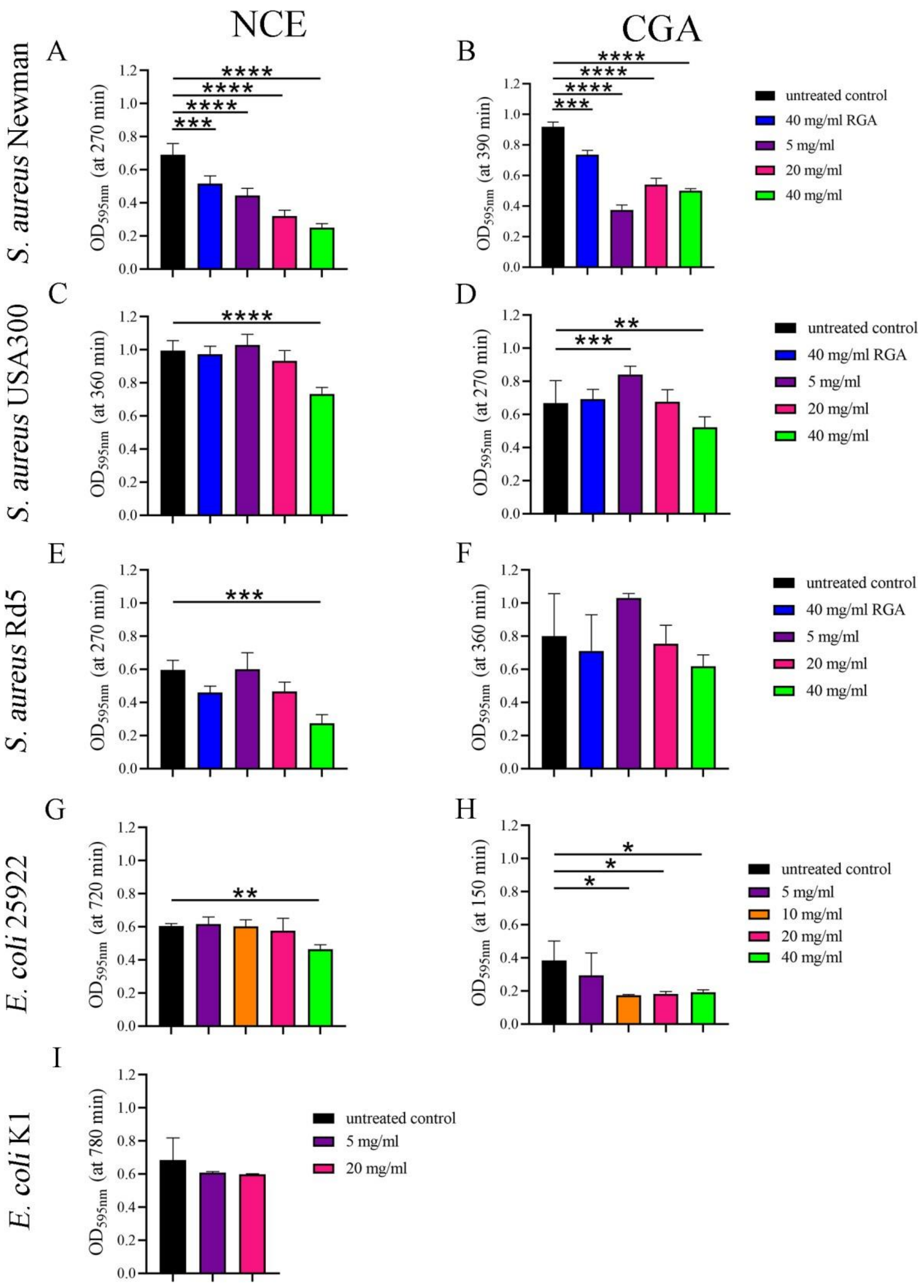

Supplementary Figure 2. Selected time points from growth curves presented in Figure 1 are depicted. The time points were selected based on the most significant difference in growth. Data are shown as mean ± SD. Statistical analysis was done with one-way ANOVA followed by Dunnett's multiple comparisons test between untreated control and treated samples. P values *p<0.05, **p< 0.01, ***p< 0.001 and ****p< 0.0001 were considered significant.

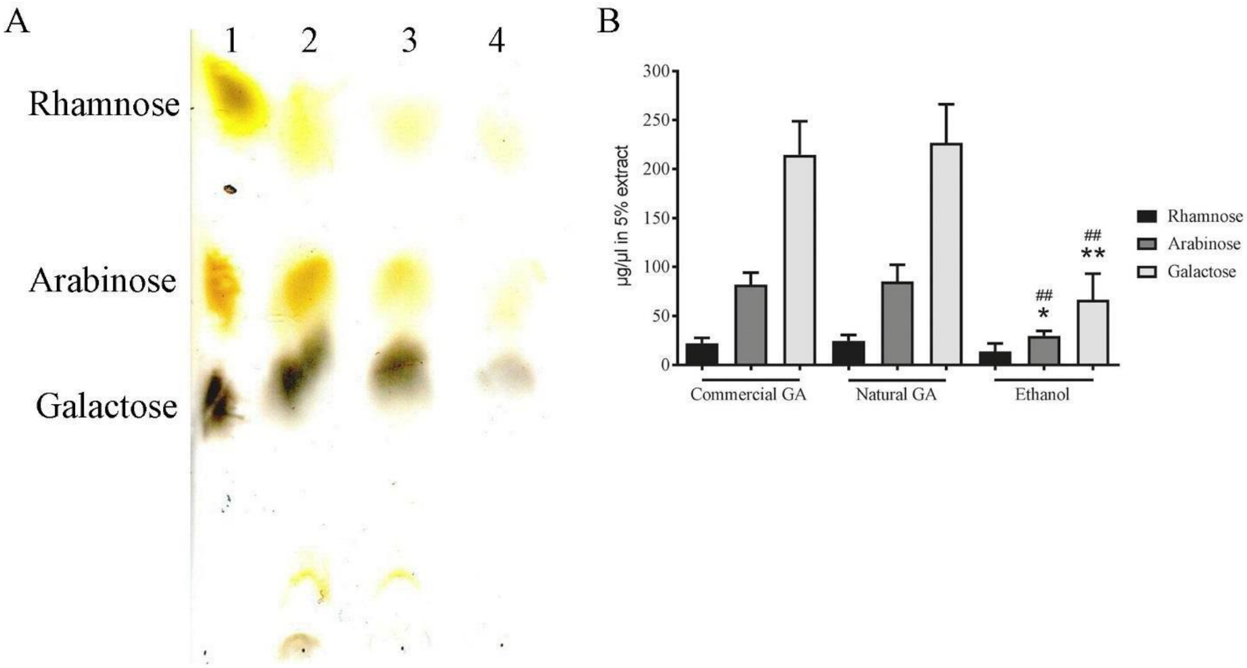

Supplementary Figure 3. (A) TLC plate revealed monosaccharide composition of CGA (A2), NCE (A3) and EP (A4) after hydrolysis with trifluoroacetic acid. Rhamnose, arabinose and galactose (main sugar in GA) were used as control (A1). (B) CP ATLAS software was used for quantification of monosaccharaide composition, CGA and NCE showed similar percentage of monosaccharide composition but EP contains significant less galactose and arabinose compared to NCE and CGA. Data are shown as mean ± SD. Statistical analysis was done by one-tailed, unpaired Student's t-Test between commercial GA (#) and ethanol and natural GA and ethanol (*). P values *p<0.05 and **p< 0.01 were considered significant.

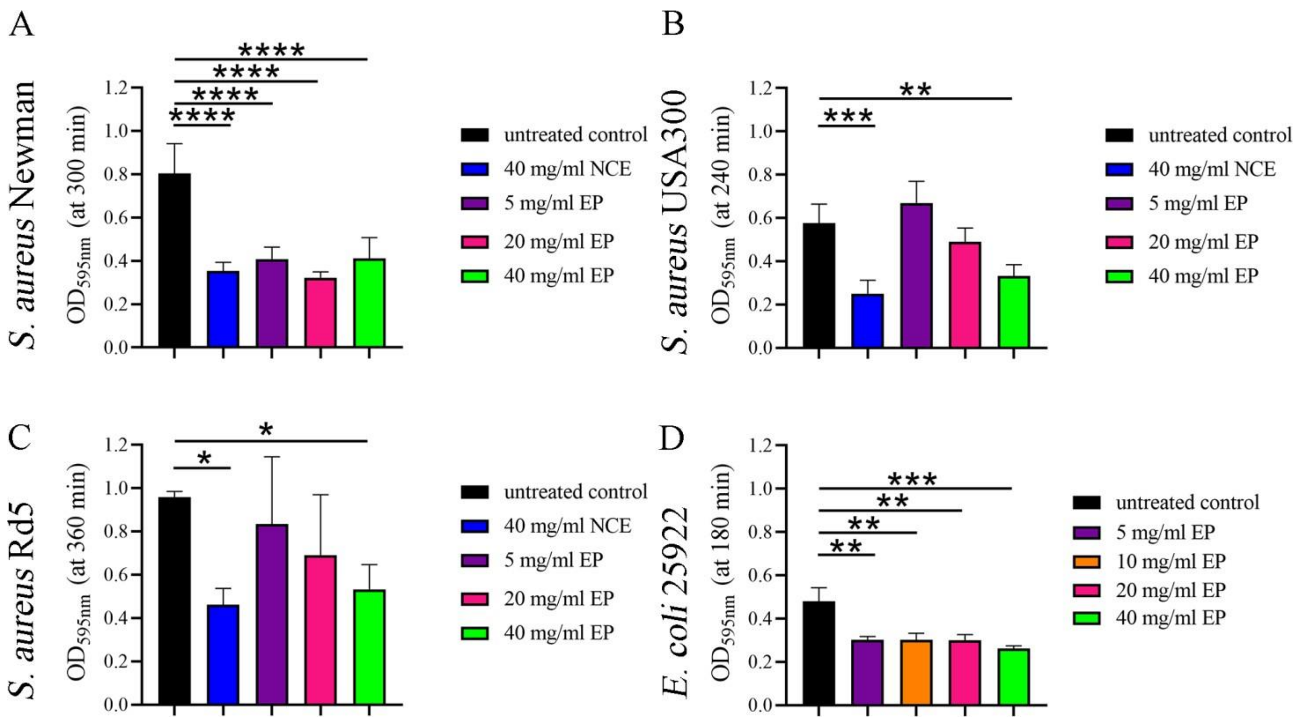

Supplementary Figure 4. Selected time points from growth curves presented in Figure 2 are depicted. The time points were selected based on the most significant difference in growth. Data are shown as mean ± SD. Statistical analysis was done with one-way ANOVA followed by Dunnett's multiple comparisons test between untreated control and treated samples. P values $*p<0.05$, $**p< 0.01$, $***p< 0.001$ and $****p< 0.0001$ were considered significant.

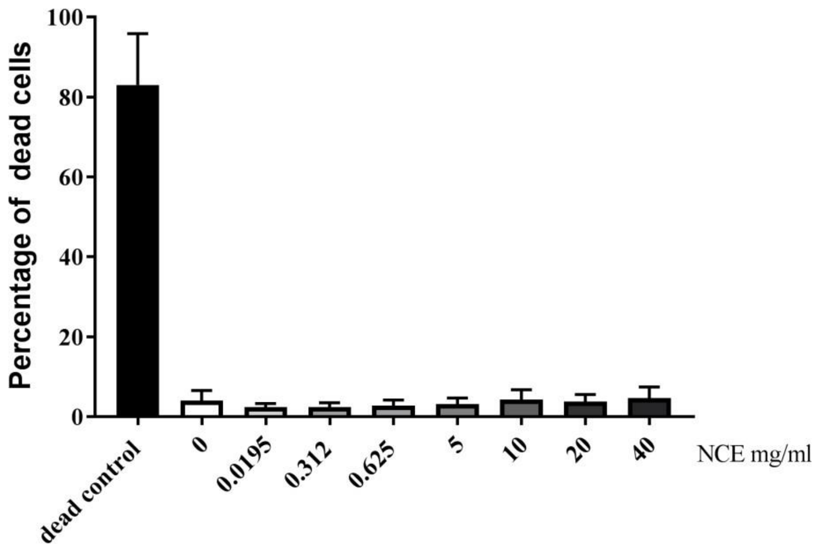

Supplementary Figure 5. No cytotoxic effect of NCE on bovine granulocytes after 120 min. Bovine granulocytes were incubated with different NCE concentrations. As dead control cells were treated with 0.2% Triton X-100. Untreated cells were used as negative control. Percentages of dead cells were determined by FACS measurement after staining cells with propidium iodide. NCE has no cytotoxic effect on granulocytes in the concentration range between 0.0195 mg/ml to 40 mg/ml. Statistical significance was tested with unpaired ordinary one-way ANOVA compared to untreated control followed by Dunnett's multiple comparison test. Dead control was excluded in analysis. Analysis was done n=4 or n=7, respectively. Data are shown as mean ± SD.

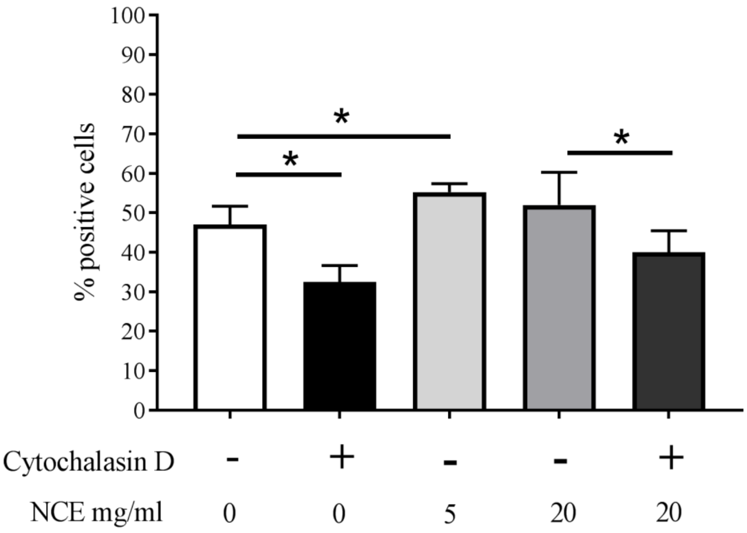

Supplementary Figure 6. NCE induces attachment and uptake of *S. aureus* bio particles by bovine granulocytes after 1 h of incubation. The association/uptake of *S. aureus* bioparticles was analysed by FACS after treatment with NCE and compared to untreated cells. The percentages of associated positive singlet cells were analyzed using FlowJo software V10. 5 mg/ml NCE significantly increase uptake compared to untreated cells. As control, cytochalasin D was used to block phagocytosis and significantly inhibited association/uptake in NCE treated cells and untreated cells. Data were analyzed with one-tailed paired Student's t-test, *p < 0.05 considered significant, data presented of n=3 independent experiments. Data are shown as mean ± SD.

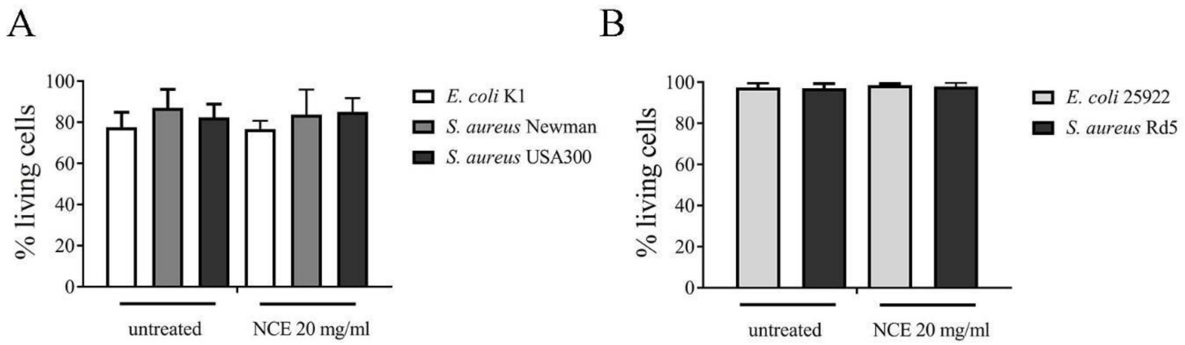

Supplementary Figure 7. Viability of immune cells in gentamicin and vancomycin protection assay using trypan blue. (A) Percentage of living human immune cells (n=4) (B) Percentage of living bovine immune cells (n=3). No cytotoxic effect on immune cells was detectable after incubation with 20 mg/ml NCE. Data are shown as mean ± SD. No significant difference was found with ordinary one-way ANOVA analysis.

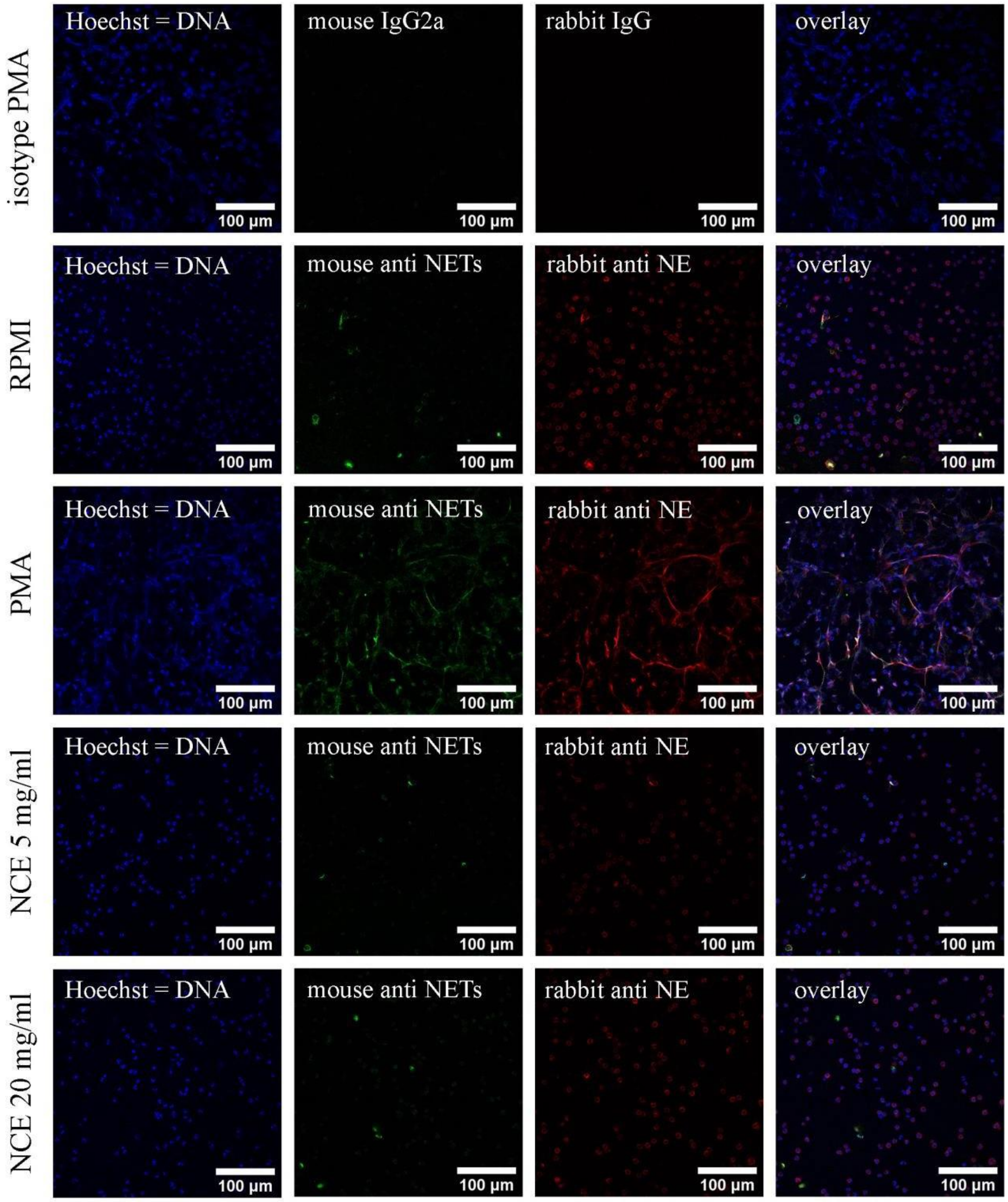

Supplementary Figure 8. Representative pictures for NET formation after stimulation of human granulocytes with 5 mg/ml and 20 mg/ml NCE. Formation of NETs was analyzed with immunofluorescence microscopy. After 4 h of incubation at 37°C and 5% CO_2 the cells were fixed. Afterwards NET staining for immunofluorescence microscopy was conducted (blue = DNA [Hoechst], green = DNA/histone-1-complexes [NETs] and red=neutrophil elastase). NCE did not induce NET formation as seen in representative pictures compared to positive control (PMA) and an unstimulated control (RPMI). The microscope settings were adjusted based on the isotype control. Representative pictures are presented. Each sample was conducted in duplicates with a total of 6 randomly selected pictures (n =1).
